# Supplementary material for: Dopamine neuron activity encodes the length of upcoming contralateral movement sequences
Source: Curr Biol. 2024 Mar 11;34(5):1034–1047.e4. doi: 10.1016/j.cub.2024.01.067 (PMC10931818; doi:10.1016/j.cub.2024.01.067)
Supplement: Document S1. Figures S1–S5 and Table S1 [file mmc1.pdf]

Current Biology, Volume *34*

Supplemental Information

Dopamine neuron activity encodes the length of upcoming contralateral movement sequences

Marcelo D. Mendonça, Joaquim Alves da Silva, Ledia F. Hernandez, Ivan Castela, José Obeso, and Rui M. Costa

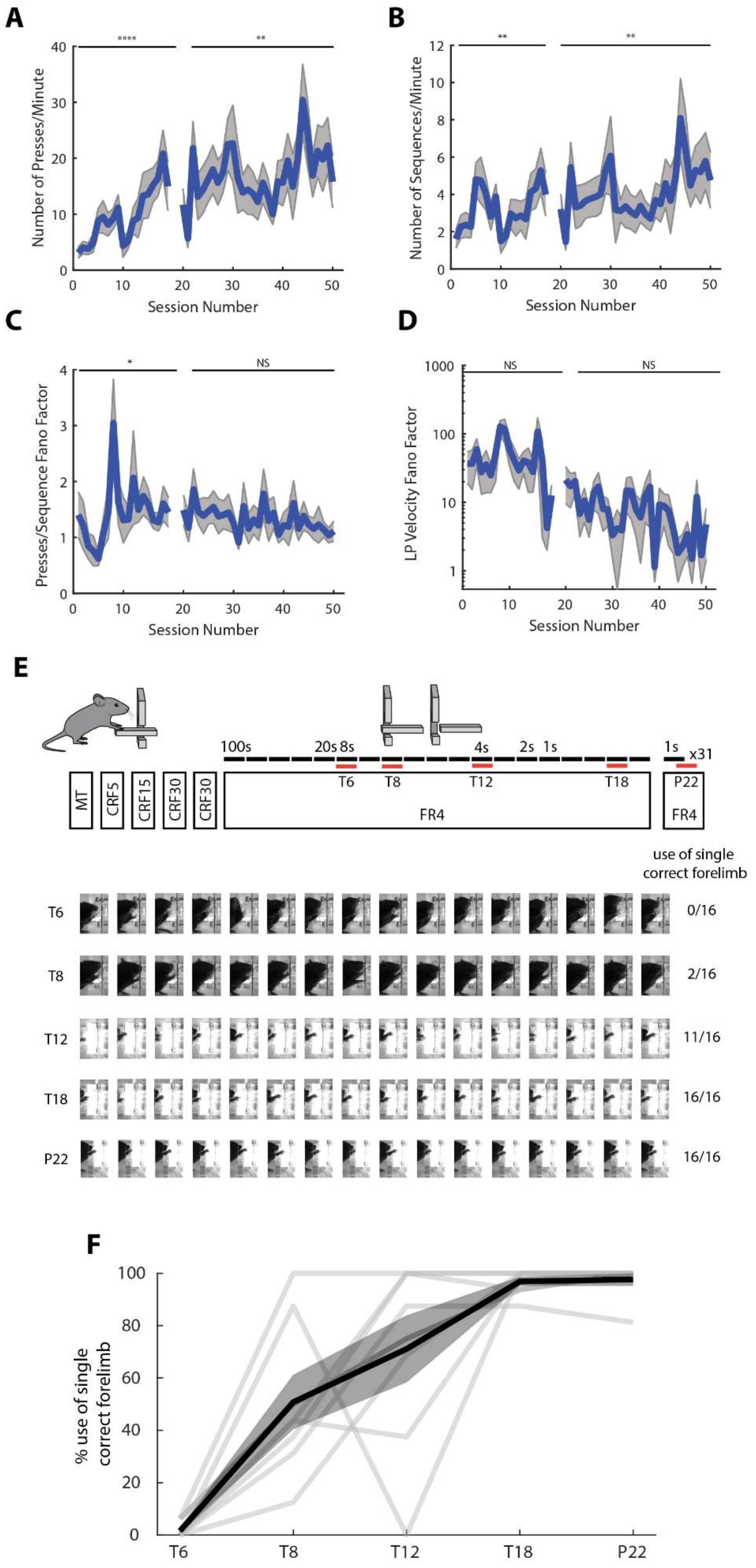
A
B
C
D
E
F
Number of Presses/Minute
Number of Sequences/Minute
Presses/Sequence Fano Factor
LP Velocity Fano Factor
Session Number
****
**
*
NS
100s
20s
8s
4s
2s
1s
x31
T6
T8
T12
T18
P22
MT
CRF5
CRF15
CRF30
FR4
use of single
correct forelimb
0/16
2/16
11/16
16/16
% use of single
correct forelimb

Figure S1. A task for assessment of single individual forelimb movements, Related to Figure 1. A) Number of lever presses/minute. **B)** Number of performed sequences/minute. **C)** Fano Factor of the number of presses/sequence **D)** Fano Factor of the average velocity of lever press. Details on Table S1. **E)** Frames corresponding to the moment of lever press were identified for 5 specific session of the training schedule (Training days – T6, T8, T12 and T18 and Performance session – P22). Sixteen (16) randomly selected frames corresponding to 16 lever presses (or all, if less) were subsampled and visually inspected to assess the use of the correct forelimb. Data from one example animal is provided. **F)** Data from the 8 mice is summarized. In the last sessions near all presses (97.66% ± 2.34) were performed by the correct forelimb.

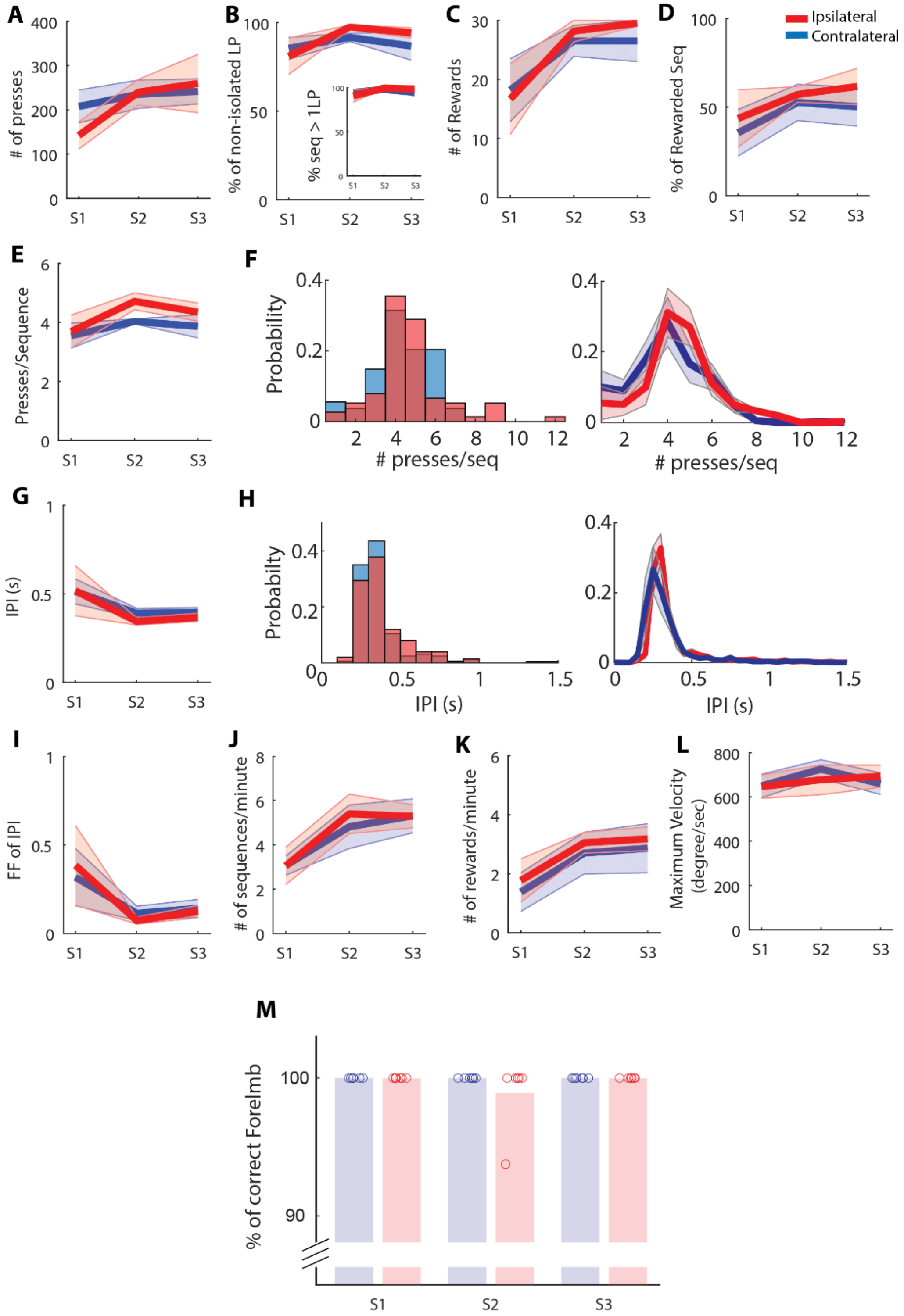

Figure S2. Mice perform the task using either the ipsi and contralateral forelimbs, Related to Figures 2, 3 and 4. Behavioral results from the 6 mice used in the analysis described in Figures

2-4. Data from sessions included in analysis in Figure 2 and Figure 3. Statistical details on Table S1. **A)** Total number of presses/session, **B)** % of sequences composed by more than one lever press - Inset % non isolated lever presses **C)** Number of Presses/Sequence. **D)** Histogram of the distribution of number of presses/sequence for ipsi and contralateral movements for one example animal (left) and for all animals across one day (right). **E)** Number of Rewards/Session **F)** % of rewarded Sequences. **G)** InterPress Intervals. **H)** Histogram of the distribution of IPIs for ipsi and contralateral movements for one example animal (left) and for all animals across one day (right) **I)** Fano Factor of InterPress Intervals **J)** Number of sequences performed/minute **K)** Number of rewards obtained/minute. **L)** Maximum velocity per press. **M)** % of correct forelimb use.

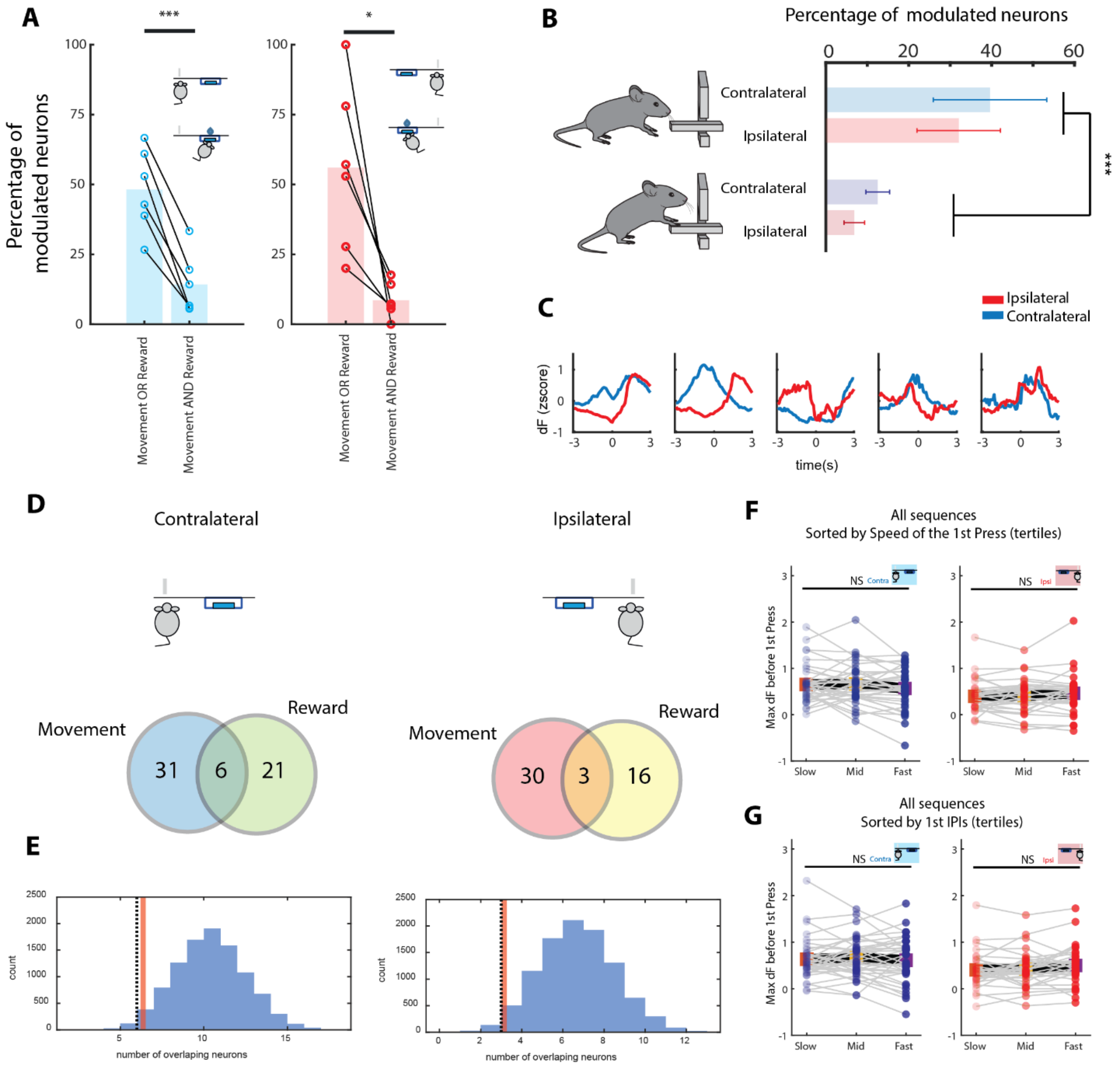

Figure S3. Modulation of activity of DANs by different events and considering different behavioral dimensions, Related to Figures 2 and 3. A) SNc DANs are modulated by one event. Neurons are more commonly modulated by only one event (movement or reward) than the two events (Left: 48,17 ± 6.07 vs. 14.21 ± 4.45, t=8.743, df=5, p<0.001; Right: 55.99 ± 12,28 vs 8.58 ± 2.60, t=3.576, df=5, p=0.0159) **B)** Activity before movement onset is more common that during execution. Number of neurons whose modulation started before movement sequence initiation (compare with Figure 2) and during sequence execution. Two-way repeated-measures ANOVA; main effect before/after F(1,5)=17.33, p=0.0088; main effect side F(1,5)=0.3986, p=0.5555; interaction effect F(1,5)=0.008, p=0.9336 **C)** Example of matched ROIs aligned to first lever press when action is performed by contralateral and ipsilateral forelimb. **D)** Overlap between reward-modulated neurons and movement initiation neurons is minimal and lower than expected by random allocation. **E)** Monte Carlo simulations (10,000 samples) were used to generate a distribution of the number of overlapping neurons for first press and reward, assuming random

assignment. Red line denote the lower margin of the one-sided 95% confidence interval of this simulation (upper limit is $+\infty$). Dashed line represents the number of overlapping neurons found in our experiments. **F)** Activity of movement modulated neurons, sorted by tertiles of the speed of the 1st press in the sequence for either ipsi ($F(2,72)= 1.557$, $p=0.2178$) or contralateral ($F(2,64)=1.127$, $p=0.335$) conditions. **G)** Activity of movement modulated neurons, sorted by tertiles of the duration of the 1st Interpress Interval for either ipsi ($F(2, 72)=0.3528$, $p=0.7039$) and contralateral ($F(2,64)=1.941$, $p=0.1519$) conditions.

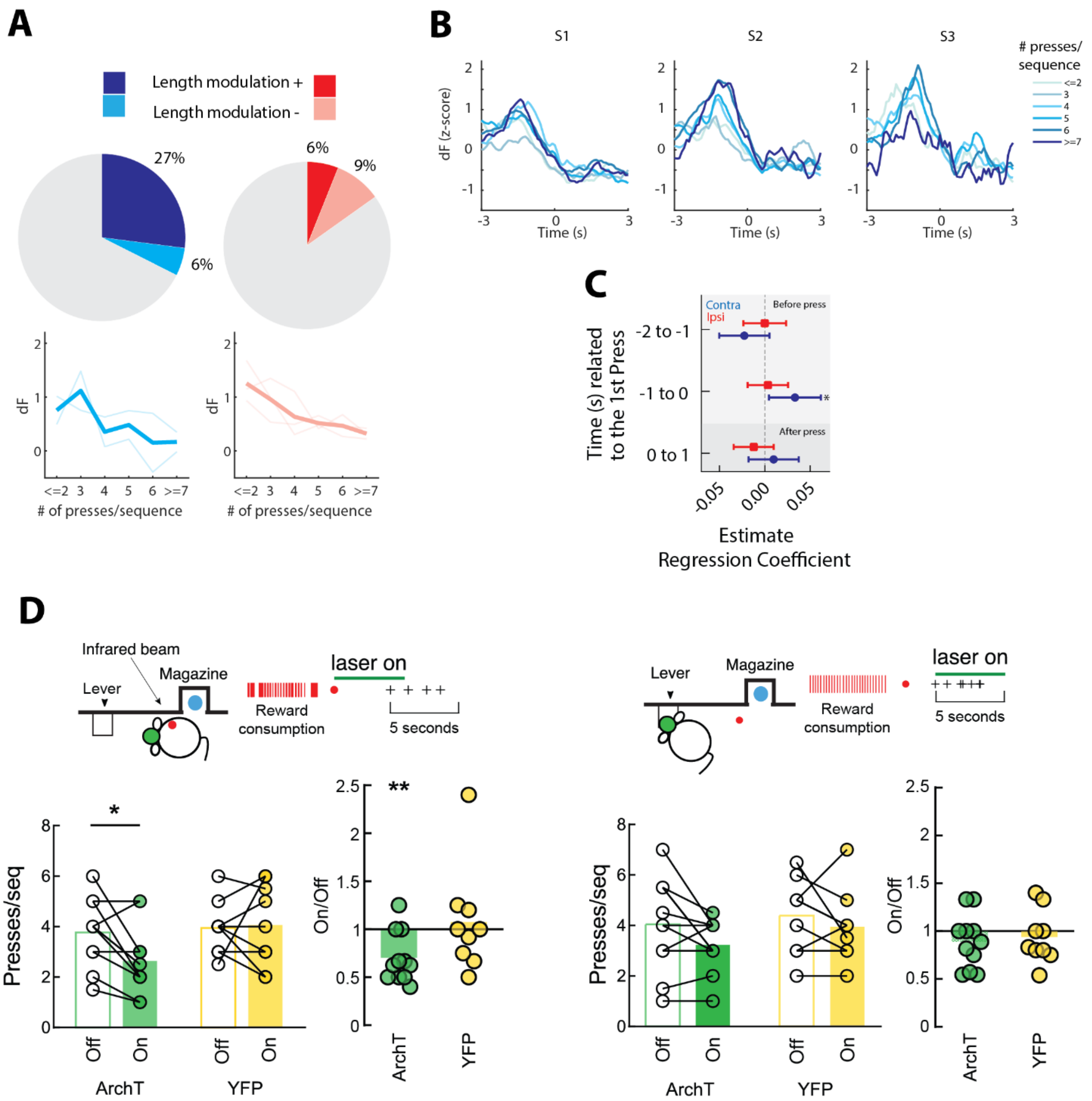

Figure S4. Transient SNc activity before the first lever press encodes the length of contralateral movement sequences, Related to Figures 3 and 5. A) Number of negatively modulated neurons in the ipsi and contralateral conditions. **B)** Activity of one example neuron (the one presented in Figure 3B) in Session 1, 2 and 3 sorted by number of presses/sequence in the contralateral sequence performance. **C)** A set of linear regression models were built to predict the number of presses based on trial-by-trial neural activity (adjusted for the neuron and mouse identity). When activity is considered in a trial-by-trial basis, only activity 1 second before first press of the contralateral sequences is related to the sequence length (R^2=0.158). **D)** Optogenetic inhibition of SNc DA neurons before movement initiation leads to a reduction in the length of movement sequences in a FR8 task. Left – Inhibition before sequence initiation leads to a

significant reduction in the number of presses in sequence (planned comparison ON/OFF ArchT: t(10)=3.023, p=0.0128; YFP: t(8)=0.2088, p=0.8398). When light-ON results were normalized to light-OFF results, there was a trend to a difference between groups: U=24, p=0.0502, with a significant difference from 1 (no change) in the ArchT group: t(10)=3.707, p=0.004, but not in the YFP group (t(8)=0.4123, p=0.6909). If inhibition was started after first press, no effect was seen neither in ArchT mice (t(10)=2.043, p=0.0683) or YFP mice (t(8)=0.9363, p=3765). When the results were normalized, there was no difference between groups (U=46.50, p=0.8371) and neither groups was significantly different from 1 (ArchT: t(10)=1.300, p=0.2229, YFP: t(8)=0.6517, p=0.5329)

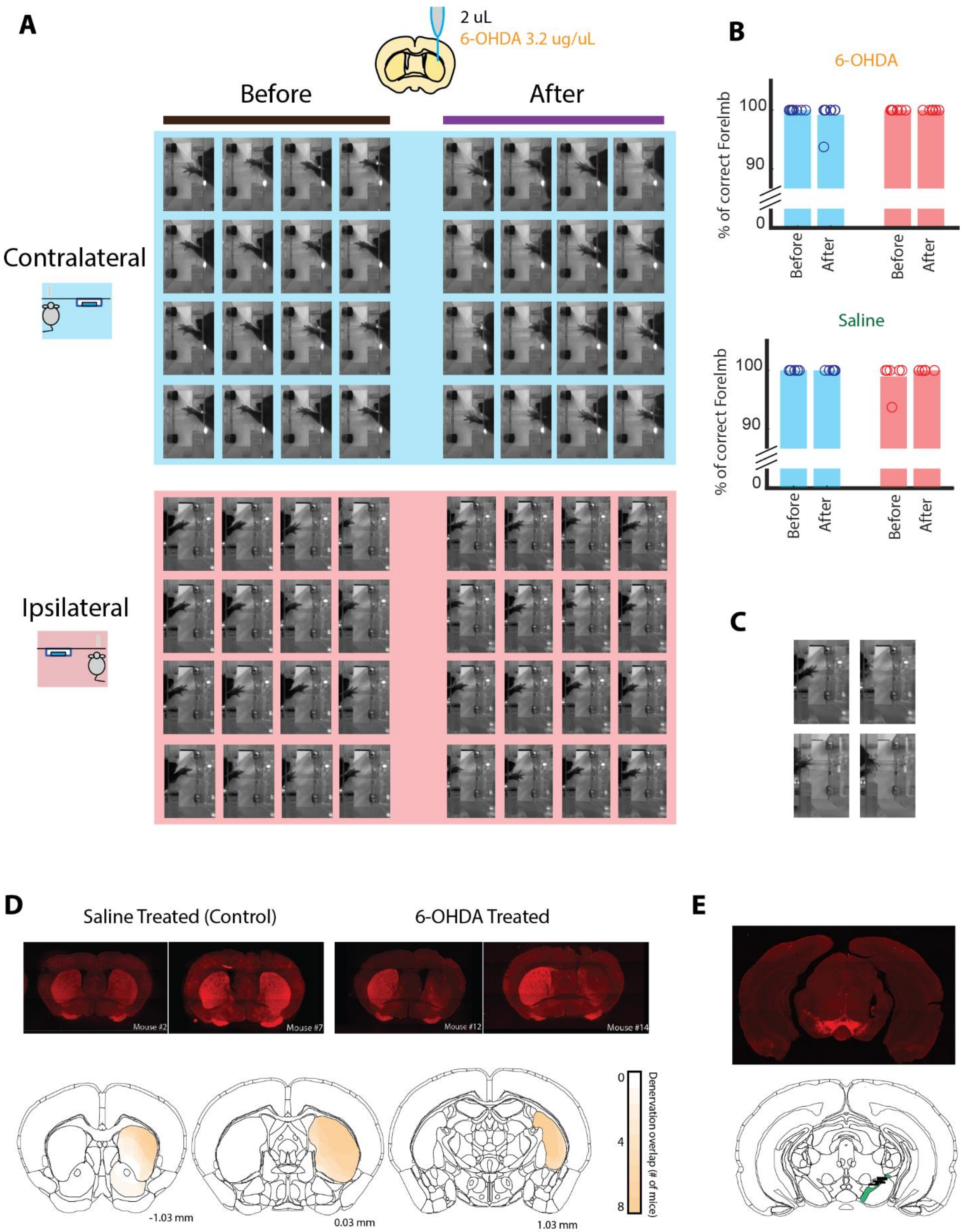

Figure S5. Mice solve the task using the forelimb contralateral to 6-OHDA lesion, **Related to Figure 5. A)** Still images from randomly selected lever presses of an example 6-OHDA treated mouse. Sixteen (16) randomly selected lever lever presses are used as an example in the 4

conditions (ipsi/contralateral forelimb and before/after 6-OHDA lesion). The mouse used the experiment-intended forelimb in all situations. **B)** For each condition and each animal 16 stills from randomly selected lever presses were visually inspected to assess the use of the correct. Two mice performed 1/16 (6.25%) of the inspected lever presses/condition with the incorrect forelimb (one 6-OHDA treated mouse in the contralateral forelimb after lesion and one saline treated mouse in the ipsilateral forelimb before). Percent of usage of the correct forelimb did not significantly changed after 6-OHDA lesion (Contra: 100% ± 0% to 99.21% ± 0.78; Ipsi: 100% ± 0 to 100% ± 0) or saline injection (Contra: 100% ± 0% to 100% ± 0; Ipsi: 98.96% ± 0.90 to 100% ± 0). **C)** The two situations with incorrect forelimb used are represented on the right images. An example of the same mouse using the correct forelimb is provided for comparison (left). **D)** Top Left: Tyrosine Hydroxylase staining of 2 example mice treated with 2 uL of intrastriatal saline (control group of Figure 5). Top Right: Tyrosine Hydroxylase staining of 2 example mice treated with 2 uL (3.2 ug/uL) of intrastriatal 6-OHDA (lesioned group in Figure 5). Bottom: Schematics representing overlap of 6-OHDA lesion across the 8 mice. Images were rotated as necessary to display the overlap of lesions in the same hemisphere (part of the animals were treated in the left striatum and the other part in the right). **E)** Tyrosine Hydroxylase staining of one of the mice with lens implant (left). Location of implanted lenses in 5 mice. Bars denote approximate position of lens tip. Lens trajectory was not possible to properly assess in one mouse due to severe tissue damage during perfusion.

Fig	Sample size (n)	Statistical tests	Value
1B	Wild-type (n=8)	One-way repeated measures ANOVA	Learning: **** F (18, 126) = 4,536 p<0.0001
			Performance: F (30, 210) = 1,174 p=0.2544
1C	Wild-type (n=8)	Mixed Effects	Learning: **** F (18, 109) = 7,526, p<0.0001<br>Learning Inset: **** F (18, 109) = 8.856, p<0.0001
			Performance: F (30, 200) = 1,283, p=0.1605<br>Performance Inset: F (30, 200) = 1,359, p=0.1121
1D	Wild-type (n=8)	Mixed Effect	L: F (18, 109) = 0.7912, p=0.7065
			P: * F (30, 200) = 1.642, p=0.0247
1E	Wild-type (n=8)	Mixed Effect	L: **** F (18, 109) = 10.52 p<0.0001
			P: F (30, 200) = 1,191, p=0.2383
		One sample t-test (vs. 4)	t7=0.5169, p=0.6212
1I	Wild-type (n=8)	Mixed Effect	L: ** F (18, 108) = 2,134, p=0.0089
			P: F (30,207) = 1.015, p=0.4507
		One sample t-test (vs. 1/3)	t7=0.3619, p=0.7281
1J	Wild-type (n=8)	Mixed Effect	L: F (18, 108) = 0.8126, p=0.6819
			P: * F (30, 200) = 1.736, p=0.0142
1K	Wild-type (n=8)	Mixed Effect	L: **** F (18, 109) = 3,250. p<0.0001
			P: F (30, 200) = 1.309, p=0.1423
2J	% of Positively modulated neurons (n=6)	Paired t-test	t=0.357, df=5, p=0.7356
2K	Positively modulated neurons:<br>Contra: n=37<br>Ipsi: n=33	Unpaired t-test	t=2.014, df=68, *p=0.0480
3A	Positively modulated neurons. Contra: n=37: Ipsi: n=33	Mixed effects analysis<br>Fixed effect<br>Test for Linear trend<br>Slope of linear fit	Contra:<br>F (5, 168) = 4.235, p=0.0012<br>**** F (1, 168) = 20.65, p<0.0001<br>Slope (95% CI): 0.0639 (0.0361 – 0.0916)<br>Ipsi:

			F (5, 151) = 1.470, p=0.2030<br>F (1, 151) = 0.4521 p=0.5023<br>Slope (95% CI): -0.0086 (-0.0347 – 0.0170)
3C	Length modulated neurons.	Fisher Exact test	* Contra: n=10/37: Ipsi: n=2/33; p=0.0266
3F	ROIs correlation n=114	One-way repeated measures ANOVA<br>Tukey Post-hoc test	F(1.353, 152.8) = 155.1 p<0.0001<br>*** Matched vs. Different: p<0.001<br>*** Matched vs. Shuffled: p<0.001
3G	Positively modulated neurons. Contra: n=37: Ipsi: n=33	Mixed effects analysis<br>Fixed effect<br>Test for Linear trend<br>Slope of linear fit	Contra:<br>F (5, 175) = 6.207, p<0.0001<br>**** F (1, 175) = 24.69, p<0.0001<br>Slope (95% CI): 0.0607 (0.0366 – 0.0848)<br>Ipsi:<br>F (5, 157) = 1.038, p=0.2913<br>F (1, 157) = 1.202 p=0.2470<br>Slope (95% CI): 0.0112 (-0.0099 – 0.0345)
3H	Length modulated neurons.	Fisher Exact test	* Contra: n=9/37: Ipsi: n=1/33; p=0.0151
4C	% of Positively modulated neurons (n=6)	Paired t-test	t=0.2668, df=5, p=0.8003
4F Left	% of Positively modulated neurons (n=6)	Paired t-test	t=0.1452. df=5, p=0.8902
4F Right	Positively modulated neurons<br>Contra: n=27<br>Ipsi: n=19	Unpaired t-test	t=0.974, df=44, p=0.3355
4G Left	% of Positively modulated neurons (n=6)	Paired t-test	t=0.1156, df=5, p=0.9125
4G Right	Positively modulated neurons<br>Contra: n=12<br>Ipsi: n=22	Unpaired t-test	t=2.723, df=32, p=0.0104
5C left	Presses/sequence (n=8)	Repeated measures two-way ANOVA	**** Time: F (1, 7) = 68.90 P<0.0001<br>Forelimb: F (1, 7) = 4.704 p=0.0667

			* Time x Forelimb: F (1, 7) = 11,11 p=0.0125<br>Sidak's multiple comparison test<br>*** Contralateral p=0.0005<br>Ipsilateral p=0.1380
5C right	Normalized presses/sequence (n=8)	Paired t-test	** t=3,759, df=7 p=0.0071
		One sample t-test	**** Contralateral: t=11.07, df=7 p<0.0001<br>Ipsilateral: t=2.281, df=7 p=0.0565
5D left	Presses/sequence (n=6)	Repeated measures two-way<br>ANOVA	* Time: F (1, 5) = 7.704 p=0.0391<br>Forelimb: F (1, 5) = 2.007 p=0.2157<br>Time x Forelimb: F (1, 5) = 0.01041 p=0.9227
5D right	Normalized presses/sequence (n=6)	Paired t-test	t=0.4441, df=5, p=0.6755
5E left	% of Long sequences (n=8)	Repeated measures two-way ANOVA	*** Time: F (1, 7) = 30.12 p=0.0009<br>Forelimb: F (1, 7) = 2.087 p=0.1918<br>*** Time x Forelimb: F (1, 7) = 32.45 p=0.0007<br>Sidak's multiple comparison test<br>*** Contralateral p=0.0003<br>Ipsilateral p=0.9725
5E right	Change in long sequences (n=8)	Paired t-test	** t=4,126, df=7 p=0.0044
		One sample t-test	**** Contralateral: t=15,46, df=7 p<0.0001<br>Ipsilateral: t=0.2100. df=7 p=0.8397
5F left	% of Long sequences (n=6)	Repeated measures two-way ANOVA	Time: F (1, 5) = 4.911 p=0.0775<br>Forelimb: F (1, 5) = 0.8281 p=0.4046<br>Time x Forelimb: F (1, 5) = 0.003268 p=0.9566
5F right	Change in long sequences (n=6)	Paired t-test	t=0.5099, df=5 p=0.6319
S1A	Wild-type (n=8)	Repeated Measures ANOVA	**** L: F (18, 126) = 4.254, p<0.0001<br>**P: F (30, 210) = 2.088, p=0.0.0025
S1B	Wild-type (n=8)	Repeated Measures ANOVA	** L: F (18, 126) = 2.569, p=0.0012<br>** P: F (30, 210) = 2.006 p=0.0025
S1C	Wild-type (n=8)	Mixed Effect	* L: F (18, 109) = 1.928, p=0.0205<br>P: F (30, 200) = 1.317, p=0.1371
S1D	Wild-type (n=8)	Mixed Effect	L: F (18, 109) = 1.692, p=0.0513<br>P: F (30, 200) = 1.082, p=0.3610
S2A	Animal (n=6)	Repeated measures two-	Session: F(2,10) = 1.330, p=0.3075

		way ANOVA	Side: F(1,5)=0.2092, p=0.6666<br>Session x Side: F(2,10)=1.290, p=0.3175
S2B	Animal (n=6)	Repeated measures two-way ANOVA	Session: F(2,10) = 1.032, p=0.3914<br>Side: F(1,5)=0.03333, p=0.8623<br>Session x Side: F(2,10)=1.094, p=0.3719
S2B (inset)	Animal (n=6)	Repeated measures two-way ANOVA	Session: F(2,10) = 1.219, p=0.3358<br>Side: F(1,5)=0.3852, p=0.5620<br>Session x Side: F(2,10)=0.8604, p=0.4521
S2C	Animal (n=6)	Repeated measures two-way ANOVA	Session: F(2,10) = 6.091, p=0.0186<br>Side: F(1,5)=0.05358, p=0.8261<br>Session x Side: F(2,10)=0.2880, p=0.7558
S2D	Animal (n=6)	Repeated measures two-way ANOVA	Session: F(2,10) = 2.179, p=0.1640<br>Side: F(1,5)=0.3660, p=0.5716<br>Session x Side: F(2,10)=0.07044, p=0.9324
S2E	Animal (n=6)	Repeated measures two-way ANOVA	Session: F(2,10) = 1.985, p=0.1880<br>Side: F(1,5)=2.919, p=0.1483<br>Session x Side: F(2,10)=0.4165, p=0.6703
S2G	Animal (n=6)	Repeated measures two-way ANOVA	Session: F(2,10) = 2.009, p=0.1847<br>Side: F(1,5)=0.3257, p=0.5929<br>Session x Side: F(2,10)=0.4805, p=0.6320
S2I	Animal (n=6)	Repeated measures two-way ANOVA	Session: F(2,10) = 1.513, p=0.2667<br>Side: F(1,5)=0.004525, p=0.9490<br>Session x Side: F(2,10)=0.9085, p=0.4340
S2J	Animal (n=6)	Repeated measures two-way ANOVA	Session: F(2,10) = 3.992, p=0.0532<br>Side: F(1,5)=0.08217, p=0.7859<br>Session x Side: F(2,10)=0.1292, p=0.8803
S2K	Animal (n=6)	Repeated measures two-way ANOVA	Session: F(2,10) = 3.912, p=0.0556<br>Side: F(1,5)=0.2349, p=0.6484<br>Session x Side: F(2,10)=0.001934, p=0.9981
S2L	Animal (n=6)	Repeated measures two-way ANOVA	Session: F(2,10) = 4.075, p=0.0508<br>Side: F(1,5)=0.03228, p=0.8645<br>Session x Side: F(2,10)=0.6672, p=0.5346

Table S1. Detailed statistical analysis, Related to Figures 1, 2, 3, 4 and 5, Figures S1 and S2.
