# Supplementary material for: Dopamine neuron activity encodes the length of upcoming contralateral movement sequences
Source: Curr Biol. 2024 Mar 11;34(5):1034–1047.e4. doi: 10.1016/j.cub.2024.01.067 (PMC10931818; doi:10.1016/j.cub.2024.01.067)
Supplement: Document S2. Article plus supplemental information [file mmc6.pdf]

Current Biology

Article

Dopamine neuron activity encodes the length of upcoming contralateral movement sequences

Highlights

- Developed a freely moving task where mice learn individual forelimb sequences
- Movement-modulated DANs encode the length of contralateral movement sequences
- The activity of reward-modulated DANs is not lateralized
- Dopamine depletion impaired contralateral, but not ipsilateral, sequence length

Authors

Marcelo D. Mendonça, Joaquim Alves da Silva, Ledia F. Hernandez, Ivan Castela, José Obeso, Rui M. Costa

Correspondence

rui.costa@alleninstitute.org

In brief

Mendonça et al. show that dopamine neuron activity preceding movement in the substantia nigra *pars compacta* encodes the length of contralateral, but not ipsilateral, movement sequences. Consistently, unilateral dopamine depletion impairs the length of contralateral, but not ipsilateral, sequences.

Mendonça et al., 2024, Current Biology *34*, 1034–1047
March 11, 2024 © 2024 The Authors. Published by Elsevier Inc.
https://doi.org/10.1016/j.cub.2024.01.067

CellPress

CellPress
OPEN ACCESS

Current Biology

Article

Dopamine neuron activity encodes the length of upcoming contralateral movement sequences

Marcelo D. Mendonça,[1,2,3] Joaquim Alves da Silva,[1,3] Ledia F. Hernandez,[4,5,6] Ivan Castela,[4,5,7] José Obeso,[4,5,6,10] and Rui M. Costa[1,8,9,10,11,*]
[1]Champalimaud Research, Champalimaud Foundation, 1400 038 Lisbon, Portugal
[2]Champalimaud Clinical Centre, Champalimaud Foundation, 1400 038 Lisbon, Portugal
[3]NOVA Medical School, Faculdade de Ciências Médicas, Universidade Nova de Lisboa, Lisbon 1169 056, Portugal
[4]HM CINAC, Centro Integral de Neurociencias AC, Fundación de Investigación HM Hospitales, Madrid 28938, Spain
[5]Center for Networked Biomedical Research on Neurodegenerative Diseases (CIBERNED), Carlos III Institute of Health, Madrid 28029, Spain
[6]Universidad CEU San Pablo, Madrid 28003, Spain
[7]PhD Program in Neuroscience, Autonoma de Madrid University, Madrid 28029, Spain
[8]Departments of Neuroscience and Neurology, Zuckerman Mind Brain Behavior Institute, Columbia University, New York, NY 10027, USA
[9]Allen Institute, Seattle, WA 98109, USA
[10]Aligning Science Across Parkinson's (ASAP) Collaborative Research Network, Chevy Chase, MD 20815, USA
[11]Lead contact
*Correspondence: rui.costa@alleninstitute.org
https://doi.org/10.1016/j.cub.2024.01.067

SUMMARY

Dopaminergic neurons (DANs) in the substantia nigra *pars compacta* (SNc) have been related to movement speed, and loss of these neurons leads to bradykinesia in Parkinson's disease (PD). However, other aspects of movement vigor are also affected in PD; for example, movement sequences are typically shorter. However, the relationship between the activity of DANs and the length of movement sequences is unknown. We imaged activity of SNc DANs in mice trained in a freely moving operant task, which relies on individual forelimb sequences. We uncovered a similar proportion of SNc DANs increasing their activity before either ipsilateral or contralateral sequences. However, the magnitude of this activity was higher for contralateral actions and was related to contralateral but not ipsilateral sequence length. In contrast, the activity of reward-modulated DANs, largely distinct from those modulated by movement, was not lateralized. Finally, unilateral dopamine depletion impaired contralateral, but not ipsilateral, sequence length. These results indicate that movement-initiation DANs encode more than a general motivation signal and invigorate aspects of contralateral movements.

INTRODUCTION

Choosing which actions to perform in specific contexts is critical for survival. It is also critical to perform these actions at the right time as well as to execute them with the appropriate vigor—the right potency, speed, length, and duration. Basal ganglia circuits, and dopaminergic signaling in these circuits, are critical for the modulation of both movement initiation and movement vigor.[1,2] Accordingly, one essential feature of Parkinson's disease (PD), which is characterized by a progressive loss of dopaminergic neurons (DANs) in the substantia nigra *pars compacta* (SNc),[3] is reduced amplitude (hypokinesia) and slowness of movements (bradykinesia).

Early studies identified that SNc dopaminergic activity was modulated during large reaching movements.[4,5] More recently, the activity of DANs was found to be transiently modulated around movement onset,[1,2,6–9] and manipulations of this activity before movement onset had an impact on the probability of movement execution and the speed of movements.[1,2] This is supported by well-established observations that chronic dopamine (DA) depletion leads to decreased amplitude, peak force, and speed of movement in PD[10–12] and also in rodents.[13–15] While many studies of vigor and PD have focused on movement force and speed, the length of movement sequences (as the number of elements composing a sequence) is also critically affected. For example, gait bouts of PD patients are characterized not only by a lower speed but also by a reduced number of steps per bout.[16] However, the relationship between the activity of DANs and the length of movement sequences remains unknown.

It has been proposed that DANs influence movement vigor by modulating the motivation to behave.[17,18] Behavioral studies in PD have revealed that the deficit in movement vigor reflects a reduced probability of committing to more vigorous actions, even when necessary for obtaining a reward.[12] However, the vigor deficits in PD are not generalized, as motor signs typically start focally on one side of the body,[19] contralateral to the most denervated SNc.[20] Similarly, unilateral DA depletion in mice leads to deficits in contraversive, but not ipsiversive, movements.[21] The striatum is involved in contralateral movements,[22–24] and DANs activity is higher when animals perform contralateral vs. ipsilateral choices.[7] Thus, dopaminergic activity

 © 2024 The Authors. Published by Elsevier Inc.
This is an open access article under the CC BY license (http://creativecommons.org/licenses/by/4.0/).

CellPress
OPEN ACCESS

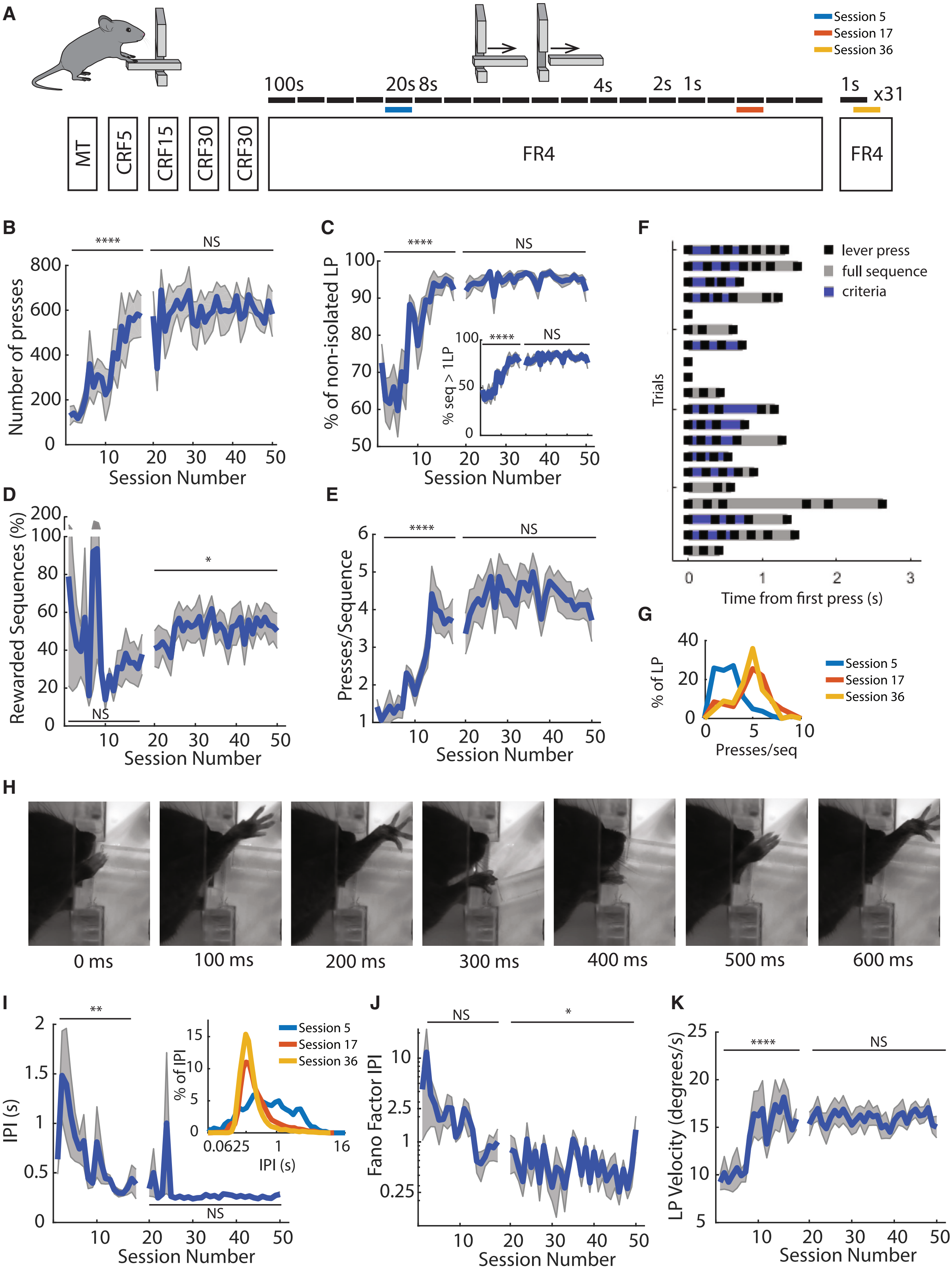

(legend on next page)

is properly placed to affect movement kinematics in a lateralized way by directly influencing medium spiny neurons' (MSNs) excitability.[25]

In this study, we investigate the hypothesis that movement-modulated DANs signal not only a general motivation to move but also invigorates aspects of contralateral movements. Toward this end, we developed a behavioral task where freely moving mice have to perform fast movement sequences using an individual forelimb to obtain reward. This paradigm allowed us to investigate movement sequences performed with either forelimb. We imaged the activity of genetically identified SNc DANs using one-photon imaging during lever-press task performance. We identified distinct populations of SNc DANs with transient activity related to movement vs. reward. Although a similar proportion of movement-modulated neurons were observed bilaterally, their activity was higher for contralaterally performed sequences than for ipsilateral ones. Furthermore, this movement-related activity was related to sequence length but only for contralateral sequences. In contrast, reward- modulated activity was not lateralized. Consistently, unilateral lesion of SNc dopaminergic neurons led to contralateral, but not ipsilateral, reduction of sequence length. These results suggest a role of dopaminergic activity before movement in invigorating the length of contralateral movements.

RESULTS

Mice learn to perform rapid single-forelimb lever-press sequences

We trained mice (n = 8) to perform a fast lever-pressing task where it was required to press a lever at increasingly higher speeds to obtain a 10% sucrose reward. During the training, spatial constraints in the lever were imposed to restrict the accessibility of the lever, forcing the animal to use only one specific forelimb (Figure 1A; Videos S1 and S2).

After introducing the animals to the apparatus and 4 days of continuous reinforcement (CRF, one press = one reward), animals were trained at a progressively faster fixed-ratio schedule (FR4, 4 presses = one reward), up to a maximum of 4 presses in less than 1 s. During the FR4 training, the lever was progressively receded to guarantee that it was only accessible to one forelimb (Figures 1A and 1H, details in the STAR Methods section). Animals were moved across a training schedule of 19 sessions, starting with FR4 in 100 s and ending with 5 sessions of FR4 in 1 s. Stability of this asymptotic performance was tested in 31 consecutive FR4/1-s sessions.

With training, the increase in the total number of lever presses (Figure 1B, $F(18,126) = 4{,}536$ $p < 0.0001$), paired the increase in the number of presses/min (reaching 14.95 ± 12.26 presses/min in the last session, $F(18,126) = 4{,}254$, $p < 0.001$ Figure S1A). During this period, animals rapidly started to organize their behavior in self-paced bouts or sequences of lever presses (Figures 1C–1G), with the percentage of lever presses performed within a sequence increasing significantly across training ($F(18,109) = 7.526$, $p < 0.0001$; Figure 1C) until there were almost no single presses occurring in isolation. In the last training session, $92.26\% \pm 8.62\%$ of lever presses occurred within a sequence of more than one lever press, and $77.12\% \pm 19.20\%$ of the movement bouts had more than one lever press (Figure 1C, inset).

The number of lever presses within a sequence progressively increased ($F(18,109) = 10.52$, $p < 0.0001$, Figures 1E and 1G), with the distribution of lever presses per sequence exhibiting a clear peak at 4 lever presses, matching the imposed rule (3.69 ± 1.71, $t7 = 0.5169$, $p = 0.6212$). Also, as time criteria become more demanding, mice decreased their inter-press intervals (IPIs) up to 0.387 ± 0.421 s (not significantly different from a target of 0.333, $t7 = 0.3619$, $p = 0.7281$, an IPI corresponding to the performance of 4 presses in less than 1 s, $F(18,108) = 2.134$, $p = 0.0089$, Figure 1I). Consistent with the reduction in the IPI, mice also increased their mean lever-press velocity with training (Figure 1K). There was also reduction in the variability of the IPIs (Fano factor, Figures 1J, $F(49,315) = 1.115$, $p = 0.2873$, post hoc test for linear trend: $F(1,315) = 16.08$, $p < 0.0001$).

After the 19 sessions of training, animals were assessed in 31 additional sessions with the criteria of 4 lever presses in less than 1 s, after behavior had reached an asymptote, with no substantial differences noted across these sessions in behavioral metrics (Figures 1 and S1; Table S1).

These data indicate that animals learned to shape their behavior to get closer to the target criteria and, after learning, this performance is stable across time. Additionally, animals can be trained to use individual forelimbs (Figures S1E and

Figure 1. A task for assessment rapid single-forelimb lever-press sequences

(A) Schematics of the training schedule. Training starts with a first session of magazine training (MT) followed by 4 sessions of continuous reinforcement schedule (CRF) where each press leads to one reward. Animals were trained for 19 sessions with an FR4 schedule of increasing time constraint (moving from 4 presses in 100 s to 4 presses in 1 s). During this period, there was also the progressive retraction of the lever that led to lever-press (LP) performance with a single forelimb. After this period, animals were moved to a performance phase comprising 31 sessions, where 4 lever presses performed in less than 1 s led to a reward. The blue, orange, and yellow lines represent example sessions to be specifically illustrated in Figures 1G and 1I. In (B), (C), (D), (E), (J), and (K), there is a discontinuation in the line. This represents the end of the stage defined as training and the beginning of the stage defined as performance.

(B and C) (B) With training, the total number of lever presses increased and (C) animals rapidly started to organize their behavior in self-paced bouts or sequences of lever presses, until there were almost no single presses occurring.

(D–G) (D) In the performance stage, about 60% of sequences were rewarded as animals reorganized their behavior (G) and started to perform lever-press sequences of 4 to 5 presses (E). (F) Example of sequences performed by a representative animal, aligned to the time of sequence initiation. Individual lever presses are marked as black ticks, the full sequence length is shaded in gray, and the IPIs that meet the session minimum target are shaded in blue.

(H) Representative frames collected from a high-speed (120 fps) camera during sequence performance.

(I) With training, the inter-press interval decreases to a mean of 0.347 s. The reorganization of IPIs distribution (inset) happens during training.

(J and K) (J) Variability of the inter-press interval decreases while press velocity increases across training (K). (Error bar denotes SEM) *$p < 0.05$; **$p < 0.01$; ***$p < 0.001$; **** $p < 0.0001$.

See also Figure S1, Table S1, and Videos S1 and S2.

CellPress
OPEN ACCESS

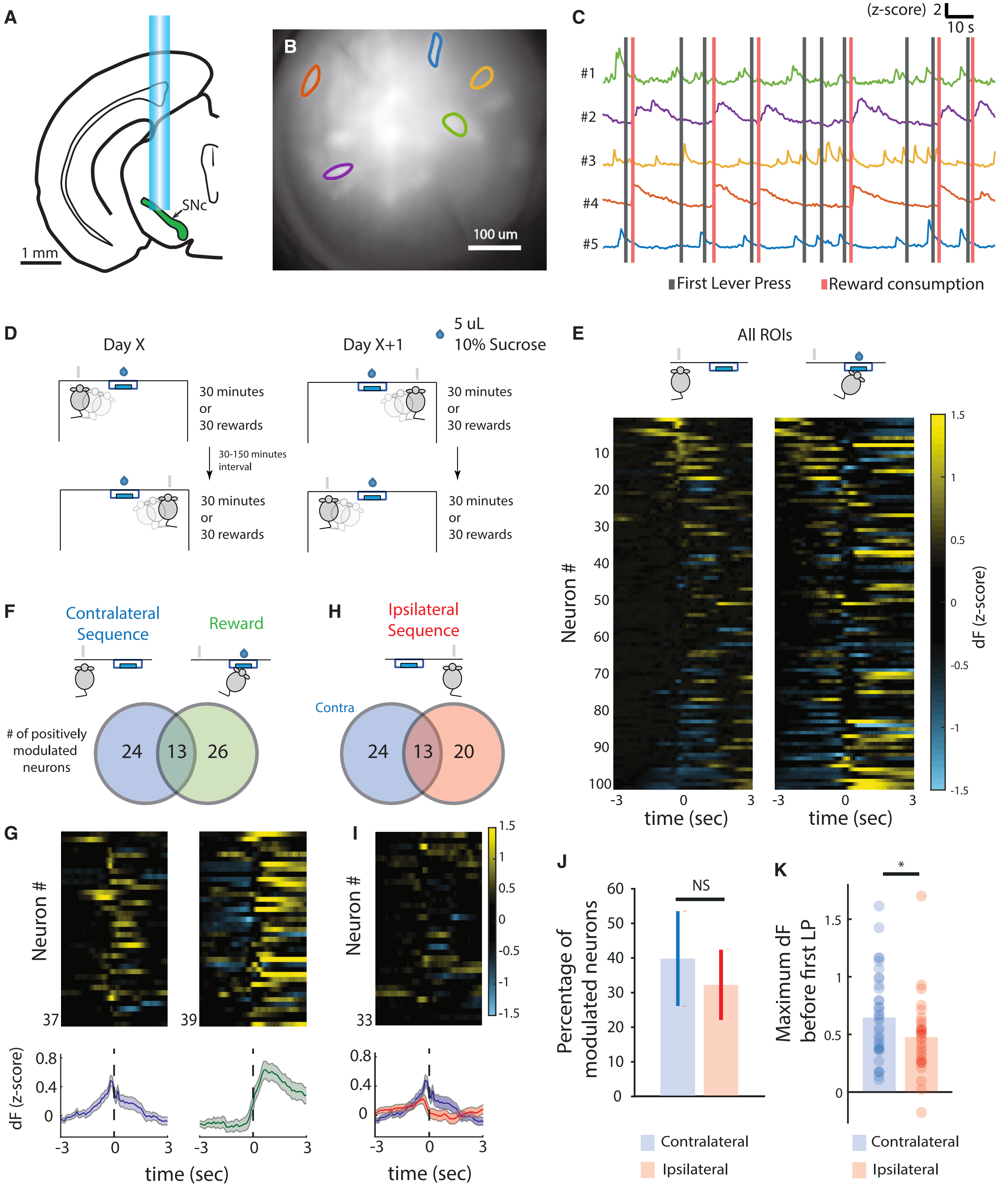

Figure 2. SNc dopaminergic neurons are transiently active before movement sequence initiation

(A) In 6 DAT-IRES:Cre mice, a miniature epifluorescence microscope was used for deep-brain calcium imaging from SNc dopaminergic neurons. Imaging was performed after animals learned the task, up to an asymptotic stage.

(B) Field of view (projection of pixel standard deviation) of a DAT-Cre mouse expressing GCaMP6f in the SNc. Regions of interest (ROIs) correspond to traces in (C).

(legend continued on next page)

S1F), allowing us to test ipsilaterally and contralaterally performed movement sequences.

Transient activity of SNc dopaminergic neurons precedes movement sequence initiation

In order to investigate the activity of SNc DANs during the execution of contralateral vs. ipsilateral movements, we chronically implanted gradient index (GRIN) lenses above the SNc (Figure S5E for lens location), injected a genetically encoded calcium indicator (GCaMP6f) into genetically identified dopaminergic SNc cells (DAT-Cre), and imaged the activity using a one-photon miniaturized epifluorescence microscope[26] (Figures 2A and 2B). Half of the animals (total n = 6 mice) had a virus injection and lens implanted in the left hemisphere and the other half in the right one. We trained these animals in the same task described above, with 2 independent sessions each day; in each session, each animal had to use a distinct forelimb (Figure 2D). Each day mice were placed in a box with a lever available on one of the sides (left or right). The session ended after the animal obtained 30 rewards or 30 min had passed. After the first session, the animals were removed from this box and placed in their home cage for a period of 30 to 150 min before being trained in a second session in the box, but with the other lever available. The order of limbs trained (left or right) was pseudo-randomized across days. Mice were able to perform movement sequences with both forelimbs and, after training, no significant differences in behavioral metrics were noted between the two forelimbs (data summarized in Figure S2 and Table S1 and presented as ipsilateral and contralateral forelimb to the implanted lens). Neural activity was assessed during the phase of asymptotic performance.

Constrained non-negative matrix factorization for endoscope data (CNMF-E)[27] was used to extract activity traces for individual neurons from the microscope video (6 mice, 101 neurons; Figures 2B and 2C). Neuronal spatial footprints and temporal activity were extracted from conjoined left and right sessions. Then, for all subsequent analyses, a normalized version (Z score) of the scaled, non-denoized version of dF (change in fluorescence) extracted by CNMF-E was performed for each of the two full sessions (left and right) independently. We created peri-event time histograms using the normalized fluorescence for the first press in any lever-press sequence and reward consumption for each experimental condition (ipsilateral or contralateral forelimb performance, Figures 2D and 2E).

In line with previous results,[2] we found that ~39% of SNc dopaminergic neurons were modulated before movement sequence initiation and a similar percentage were modulated around reward. The overlap between these 2 populations was small (~13%) and not different from what was expected by chance (Figures 2F, 2G, and S3A). Some DANs also started their modulation during sequence execution, but this number was lower than those modulated before sequence performance (Only 7%–13% of neurons modulated during performance, $F(1,10) = 17.33$, $p = 0.009$; Figure S3B).

Movement-initiation neurons displayed transient increases in activity before both contralateral and ipsilateral forelimb movement sequences (Figures 2H, 2I, and S3C). However, although a similar proportion of movement-initiation neurons were active before contralateral and ipsilateral movements (39.6% ± 13.7% vs. 32.1% ± 10.01% $p = 0.7356$, paired t test, Figure 2J), the magnitude of the activity of these neurons was significantly higher in the contralateral vs. ipsilateral SNc (0.646 ± 0.061 vs. 0.427 ± 0.059, $p = 0.048$, unpaired t test, Figure 2K). This difference in neural activity could not be explained by a difference in the performance of the task, as no performance differences were identified between the ipsilateral and contralateral forelimb (Figure S2; Table S1).

These data show that, although movement-initiation neurons were found bilaterally, activity preceding contralateral limb movements is higher than the activity preceding ipsilateral movements.

DANs encode the length of upcoming contralateral, but not ipsilateral, forelimb movement sequences

We next investigated the relationship between neural activity before movement initiation and the length of the sequences. The magnitude of activity of movement-modulated DANs was sorted according to the number of presses in each sequence for either ipsilateral or contralateral sequences. The maximum activity preceding sequence initiation was linearly related to the number of presses/sequence performed by the contralateral (Figure 3A left, n = 37 neurons, $p < 0.001$), but not the ipsilateral, forelimb (Figure 3A right, n = 33 neurons, $p = 0.2660$). Our task was not designed to study dimensions related to the speed of presses, resulting in these dimensions having a low variability. No relationship between DA activity and press speed or inter-press intervals was identified (Figures S3F and S3G), but we cannot exclude the possibility that we are underpowered by design.

We quantified the number of neurons significantly modulated by sequence length (Figures 3B and S4). We identified a

(C) Example traces obtained using the CNMF-E algorithm during the FR4 task. ROIs #1 and #5 are examples of units modulated before first lever press—gray lines—and ROIs #2 and #4 are examples of units modulated after reward—red lines.
(D) Schematics of the training schedule used for this set of experiments. Mice were trained in a pseudo-randomized order across different training days, alternating between starting with the ipsilateral or contralateral forelimb.
(E) Activity of all recorded ROIs from one session aligned to the first contralateral lever press (left) and to the beginning of reward consumption (right).
(F) Venn diagram representing the number of contralateral first-press (left, blue color) and reward-related (right, green color) neurons.
(G) PETH of positively modulated neurons for first press and reward (bottom) and corresponding heatmaps (top).
(H) Venn diagram representing first-press-modulated neurons when action was performed by contralateral (blue, same number as in F) and ipsilateral (red) forelimb.
(I) PETH of positively modulated neurons for first press contralateral (blue) and ipsilateral (red) (bottom) and corresponding heatmap for ipsilateral neurons (top).
(J) Percentage of positively modulated neurons before first lever press per mouse, comparing contralateral and ipsilateral forelimb (NS, $p = 0.736$, paired t test).
(K) Maximum fluorescence in the 2 s before first lever press of positively modulated neurons when the action was performed with the contralateral and ipsilateral forelimb (contralateral, n = 37 neurons, ipsilateral, n = 33 neurons, *$p = 0.0480$, unpaired t test). Data are presented as mean ± SEM. *$p < 0.05$.
See also Figures S2, S3, and Table S1.

CellPress
OPEN ACCESS

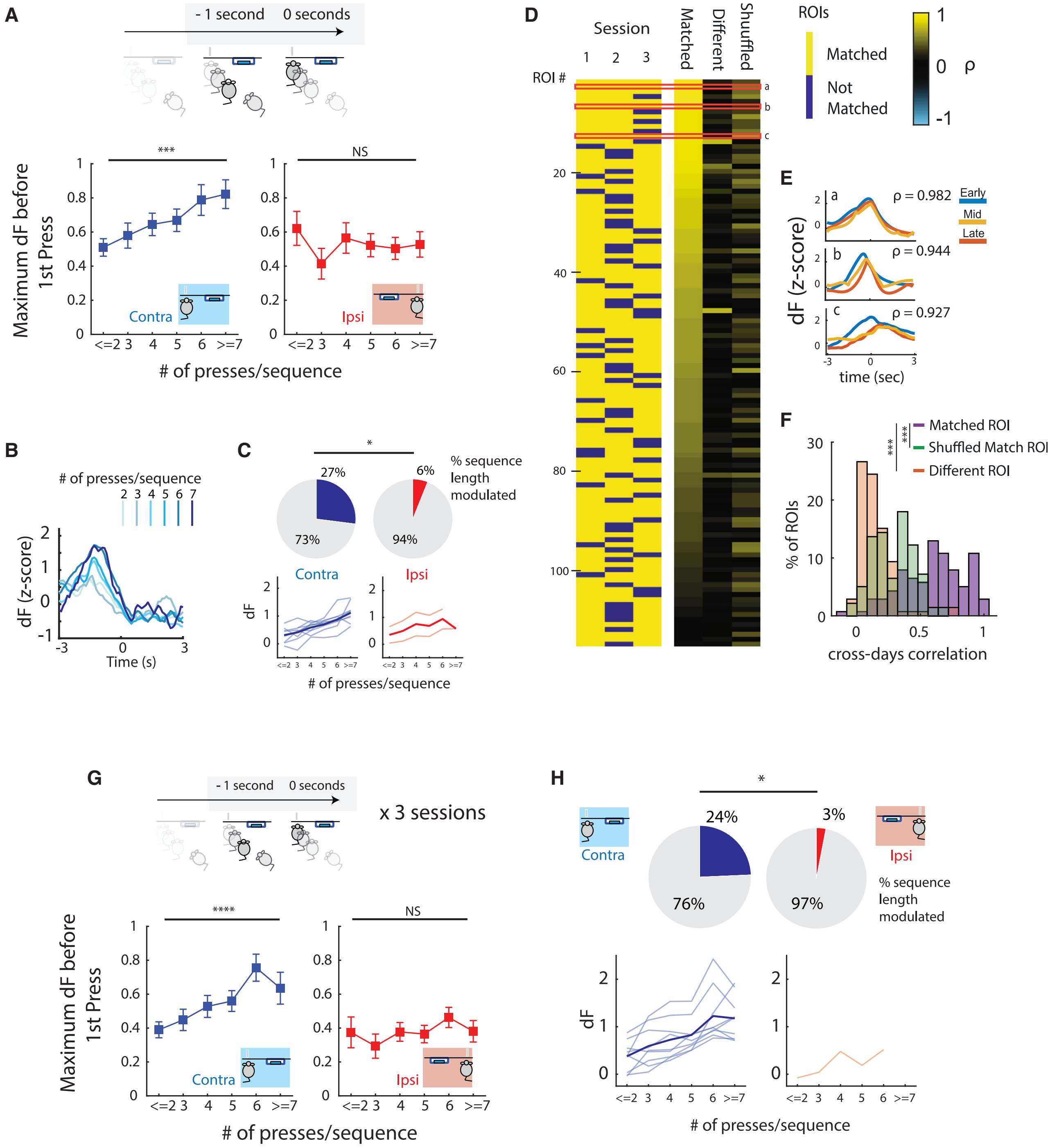

Figure 3. Transient SNc activity before the first lever press encodes the length of contralateral movement sequences

(A) Maximum fluorescence before first lever press for movement-modulated neurons, according to the number of presses/sequence performed by contralateral (left, n = 37, $F(1,168) = 20.65$, ***$p < 0.001$, test for linear trend) and ipsilateral forelimb (right, n = 33, $F(1,151) = 0.4521$, $p = 0.5023$, test for linear trend).

(B) Example of one movement-modulated neuron activity sorted by the number of presses/sequence.

(C) Percentage of neurons positively modulated by sequence length (top) in contralateral and ipsilateral conditions (*$p = 0.0266$, Fisher's exact test). Activity of individual neurons positively modulated by sequence length (light color) and average activity (strong color) in contralateral and ipsilateral conditions (bottom).

(D) Matching of ROIs identified across 3 sessions of performance. Only ROIs that were matched in at least 2 of 3 sessions were plotted (left). Heatplot showing the average cross-days correlation of matched neurons PETHs and maximum cross-days correlation of each ROI, with all ROIs of the same animal across days as a control (right). Red highlighted regions (a, b, and c) correspond to three different ROIs detailed in (E).

(legend continued on next page)

significantly higher number of length-modulated neurons contralaterally (27%, Figure 3C, left) than ipsilaterally (6%, Figure 3C, right, $p = 0.0266$). Negatively modulated neurons were scarce (Figure S4A). These data support the hypothesis that activity of a population of SNc DANs encodes contralateral sequence length.

Even accounting for some day-to-day variability in the field of view, *in vivo* calcium imaging permits us to track the same region of interest (ROI) across multiple sessions. This approach allows us to explore whether SNc neurons' activity is functionally stable across different performance sessions of highly trained movements (supporting the argument of a functional identity of specific dopaminergic neurons during task performance in a context of diversity of functions, Figure 2F). To match neurons across sessions, we used a nearest neighbor approach. For all sessions, a centroid for each ROI was calculated. For each reference centroid, distance from all centroids on the image to be compared was calculated, and the 3 ROIs with the smallest distance were visually inspected for their shape to confirm the matching. Pairing was iteratively performed across the 3 included sessions. Across the 3 sessions, we identified 114 individual ROIs that were matched in at least 2 sessions (40 ROIs—35.09%—were matched across the 3 sessions, Figure 3D)

Event-aligned activity of matched neurons was correlated across daily sessions and the results were averaged if matched in more than 2 sessions (matched group). The activity of the same neuron was correlated with the matched ROI in a different session, shuffled in time (shuffled group), and averaged across daily sessions. Correlation of activity from the reference neuron with neurons from the same animal, but not those that were spatially matched (different group), was also computed and averaged across animals. This approach revealed that there is a high correlation of event-aligned activity of spatially mapped neurons (66.7% of neurons had a strong correlation—above 0.5, Figures 3D and 3E) and that this similarity was significantly higher than correlations with non-spatially mapped ones (0.677 ± 0.049 vs. 0.147 ± 0.014 vs. 0.312 ± 0.017, repeated measures one-way ANOVA performed in the Fisher's Z transformed correlation coefficient $F(1.353, 152.8) = 155.1$, $p < 0.0001$, Tukey's test for multiple comparisons matched, vs. different, $p < 0.0001$, matched, vs. shuffled, $p < 0.0001$, Figure 3F). This analysis suggests that, in general, SNc dopaminergic neurons' functional identity remains stable across days while animals perform the learned task.

Matching across days (as performed in Figure 3E) allowed us to study the activity of the same neuron during trials from different sessions (Figure S4B). For each neuron defined as movement-modulated, we extracted the activity before movement initiation (as described in Figure 3A). Across the 3 sessions, a relationship with sequence length was present contralaterally (Figure 3G, left, $n = 37$ neurons, $p < 0.0001$) but not ipsilaterally (Figure 3H, right, $n = 33$ neurons, $p = 0.2470$). These results were also supported by a trial-by-trial analysis (Figure S4C). A significant over-representation of length-modulated neurons contralaterally was observed across the 3 sessions (24% vs. 3%, $p = 0.0181$, Figure 3I)

These data show that activity in movement-initiation SNc neurons encodes the length of contralaterally (in comparison with ipsilaterally) performed movement sequences, as neural identity keeps stable over time.

Reward-modulated DA activity is not lateralized

Modulation around reward was identifiable when mice performed the task either in the ipsilateral or contralateral condition (Figures 4A and 4B). The number of neurons was not significantly different between conditions (Figure 4C, $36.9\% \pm 5.9\%$ vs. $41.1\% \pm 14.1\%$, paired t test: $p = 0.8003$, Figure 4D). The number of reward-modulated neurons (active only after reward consumption) was similar when the action leading to that reward was performed ipsilaterally or contralaterally (~23%, Figure 4F middle, paired t test: $p = 0.8902$), and no difference was observed in the maximum fluorescence of neurons according to the side of performed action (0.95 ± 0.10 vs. 1.11 ± 0.11 Figure 4F right, t test: $p = 0.3355$). The overlap between reward-modulated neurons and movement-initiation neurons was residual (~5% of the total) and significantly lower than that which we would expect by random allocation (Figure S3D), revealing that movement initiation and reward neurons mostly represent two distinct populations.

A second group of neurons was already active before reward consumption and ramped up as animals approached the magazine (Figure 4D, bottom, hereafter called magazine-approach neurons).[28] Based on the box design, approach to the magazine could be performed with either an ipsiversive or a contraversive movement (Figure 4E). While the number of magazine-approach neurons was not significantly different during ipsiversive or contraversive movements (~15%, paired t test: $p = 0.9125$), their activity was significantly higher during contraversive movements (1.49 ± 0.21 vs. 0.66 ± 0.18 Figure 4G right, t test: $p = 0.0104$).

These data support the notion that movement-initiation- and reward-modulated neurons in the SNc are not likely the same DAN population and that neurons responsive to reward consumption do not have a lateralized representation.

Dopaminergic depletion reduces the length of contralaterally performed forelimb movement sequences

The results shown above suggest that SNc dopaminergic activity is asymmetric during single-forelimb movements and that its magnitude is related to the vigor of contralateral, but not

(E) Example PETHs of three ROIs in different sessions disclosing the similarities in average neural activity.
(F) Histogram of the cross-days correlations represented in (D). ($n = 114$ ROIs ***$p < 0.001$, post hoc Tukey's test after a repeated measures (RM) one-way ANOVA.)
(G) Neurons were matched across 3 sessions. Maximum fluorescence 1 s before first lever press for movement-modulated neurons according to the number of presses/sequence performed by contralateral (left, $n = 37$, $F(1,175) = 24.69$, ****$p < 0.0001$, test for linear trend) and ipsilateral forelimb (right, $n = 33$, $F(1,157) = 1.202$, $p = 0.2470$, test for linear trend).
(H) Percentage of neurons positively modulated by sequence length (top) in contralateral and ipsilateral conditions across 3 days (*$p = 0.0151$, Fisher's exact test). Activity of individual neurons positively modulated by sequence length (light color) and average activity (strong color) in contralateral and ipsilateral conditions (bottom).*$p < 0.05$; **$p < 0.01$; ***$p < 0.001$; **** $p < 0.0001$. Data are presented as mean ± SEM.
See also Figures S2, S3, and S4 and Table S1.

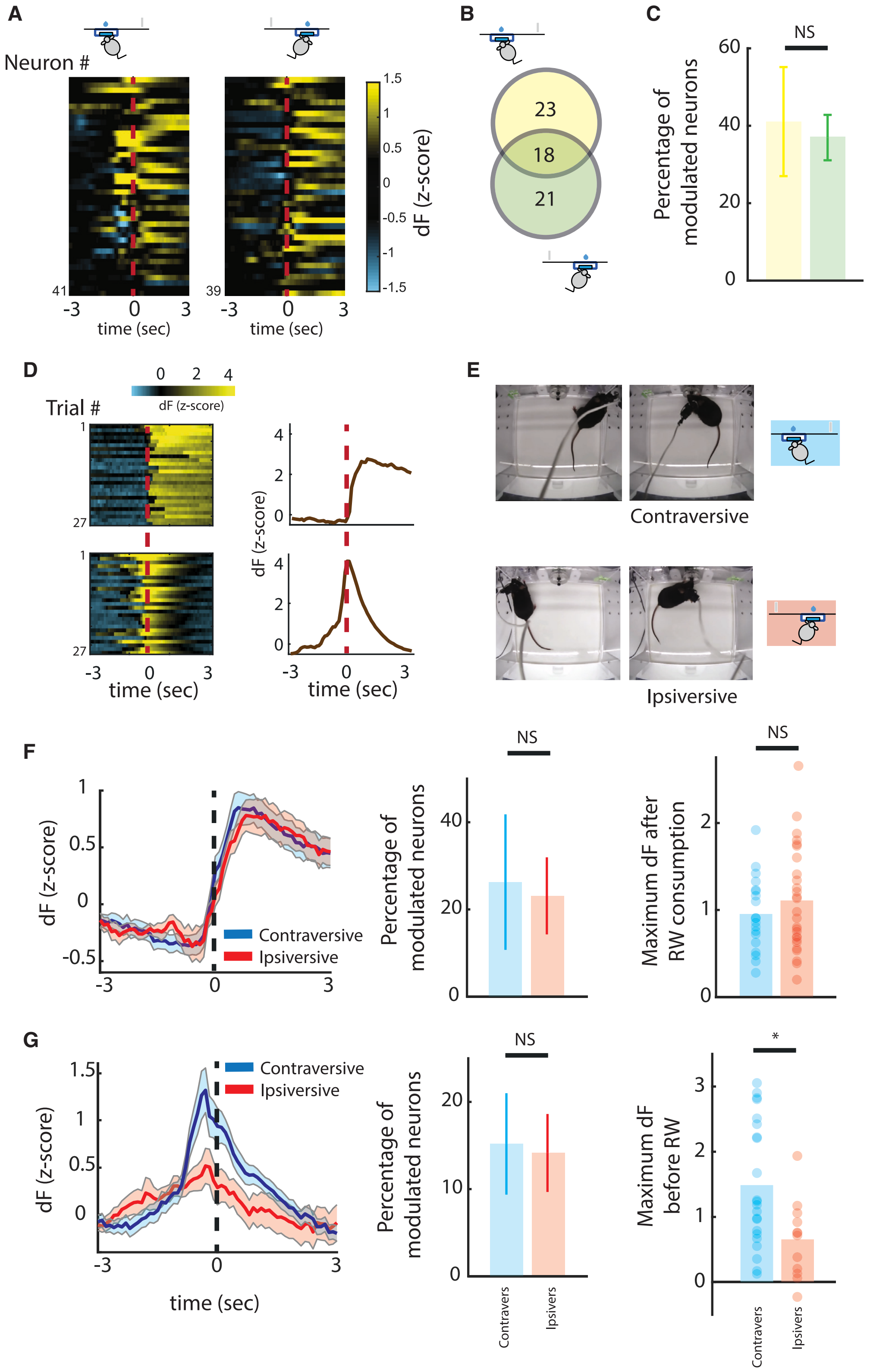

(legend on next page)

ipsilateral, sequences. We therefore tested whether unilateral loss of dopaminergic neurons in the SNc would preferentially affect the length of contralateral sequences. To achieve that, we used a unilateral striatal injection of 6-hydroxydopamine (6-OHDA), a neurotoxin that selectively affects dopaminergic neurons. It causes rapid degeneration of striatal terminals (within hours of treatment) and changes in SNc DAN markers and cell body structure and numbers are already detectable within 3 days of the lesion.[29]

A new group of 14 mice was trained in the task as described in Figure 2D until they reached an asymptotic stage (FR4/1 s). After this, using a stereotaxic approach, injection of 2 uL of 6-OHDA (n = 8) or saline (n = 6) was performed in the dorsolateral striatum in a randomly chosen side (left or right). Post-operative care was performed during the following 7 days and mice did not have access to the operant boxes. After this time period, mice were again placed in the operant boxes and had to perform the task to obtain reward using the same criteria as before treatment (FR4/1 s) (Figures 5A and 5B). Severity of DA depletion is revealed in Figure S5D.

Unilateral DA depletion led to a reduction in the number of presses per sequence (two-way repeated-measures ANOVA, time $F(1,7) = 68.90$, $p < 0.001$, forelimb $F(1,7) = 4.704$, $p = 0.0667$, time x forelimb $F(1,7) = 11.11$, $p = 0.0125$, Figure 5C left) and corresponding reduction in the percentage of long sequences (two-way repeated-measures ANOVA, time $F(1,7) = 30.12$, $p < 0.001$, forelimb $F(1,7) = 2.087$, $p = 0.1918$, time × forelimb $F(1,7) = 32,45$, $p < 0.001$ Figure 5E left). Whereas the contralateral limb to the lesion started to perform smaller sequences (4.12 ± 0.20 to 2.17 ±0,14, Figure 5C left, multiple comparison after two-way repeated-measures ANOVA, $p < 0.001$), and show a reduced number of long sequences (48.82% ± 7.72% to 8.13% ± 3.48, Figure 5E left, multiple comparison after two-way repeated-measures ANOVA, $p < 0.001$), this was not observed for the limb ipsilateral to the lesion (4.13 ± 0.34 to 3.52 ± 0.39, Figure 5C left, multiple comparison after two-way repeated-measures ANOVA, p = 0.138% and 41.34% ± 5.16% to 37.67% ± 5.67, Figure 5E left, multiple comparison after two-way repeated-measures ANOVA, $p = 0.9725$, Videos S3 and S4). Regardless of the unilateral DA depletion, mice kept performing the task with the intended limb (Figures S5A–S5C).

The relative mean sequence length (after treatment/before treatment) was significantly different between sides (Figure 5C right, paired t test, t = 3,759, df = 7 p = 0.007) and significantly different from the unit value (representing no change), but only contralaterally (Figure 5C right, one sample t test, t = 11.07, df = 7, $p < 0.001$). This result was confirmed by the significant difference in the relative proportion of long sequences between sides (Figure 5E right, paired t test, t = 4,126, df = 7, p = 0.004, significance from the unit value only identified contralaterally, Figure 5E right, one sample t test, t = 15.46, df = 7, $p < 0.001$)

By contrast, the injection of saline only led to a small and non-side-specific reduction in the average number of presses/sequence (Figure 5D left, two-way repeated-measures ANOVA, time $F(1,5) = 7.704$, $p = 0.039$, side $F(1,5) = 2.007$, $p = 0.216$, time × side $F(1,5) = 0.010$, $p = 0.923$), without a significant change in the proportion of long sequences (Figure 5F left, two-way repeated-measures ANOVA, time $F(1,5) = 4.911$, $p = 0.078$, side $F(1,5) = 0.8281$, $p = 0.405$, time × side $F(1,5) = 0.003$, $p = 0.957$). When the relative mean sequence length was compared between sides, no difference was found (Figure 5D right, paired t test, t = 0.44, df = 5, p = 0.6755), similar to the lack of a difference in sequence length change (Figure 5F right, paired t test, t = 0.5099, df = 5 p = 0.6319). 6-OHDA lesions reduce DA both before and during sequence execution. Therefore, we analyzed data from a previous study[2] and found that optogenetic inhibition of DANs before the first press of a sequence reduced the length of the subsequent sequence (in line with our observations with 6-OHDA results), but no change in sequence length was observed when inhibition started after the first press (Figure S4D). These data suggest that effect on sequence length is likely driven by a reduction in DA levels available in the striatum before, and not during, sequence performance.

Overall, these data show that unilateral DA depletion leads to a reduction in the length of contralaterally performed movement sequences without impacting the performance of ipsilateral movement sequences.

DISCUSSION

We found that activity in a subset of SNc dopaminergic neurons encodes the length of contralateral sequences—a dimension of movement vigor—before movement initiation. Using a lateralized task, we unraveled the finding that transient SNc dopaminergic activity precedes the execution of forelimb movement sequences irrespective of the limb performing the sequence.

Figure 4. Activity of reward-modulated neurons is not lateralized

(A) Heatmaps of neurons with responses around reward when animals performed the task with the ipsilateral and contralateral forelimb.

(B) Venn diagram representing these neurons when reward is collected after performing a contraversive (yellow) and ipsiversive (blue, same number as in Figure 2F) movement.

(C) Percentage of positively modulated neurons in the contraversive and ipsiversive conditions (p = 0.80, paired t test). Data are presented as mean ± SEM.

(D) Example of two neurons displaying different responses identified around reward: reward-modulated neuron (top) and magazine-approach neuron (bottom). (Left) Activity heatmap for all trials aligned to reward collection. (Right) PETH of that neuron aligned to reward collection.

(E) Top view of mouse position when pressing the lever and consuming reward in situations where it performs a contraversive (top) and ipsiversive (bottom) movement.

(F) Activity of reward-modulated neurons after a contraversive (blue) or ipsiversive (red) movement: PETH of reward-modulated neurons (left), percentage of modulated neurons per mouse (n = 6, 25.88% ± 15.40% vs. 22.80% ± 8.84%, p = 0.8902, paired t test) and maximum fluorescence after reward consumption (right) (ipsiversive, n = 27 neurons, contraversive, n = 19 neurons, p = 0.3355, unpaired t test).

(G) Activity of magazine-approach neurons after a contraversive (blue) or ipsiversive (red) movement: PETH of magazine-approach neurons (left), percentage of modulated neurons per mouse (n = 6, 15.20% ± 5.85% vs. 14.14% ± 4.47%, p = 0.9125, paired t test), and maximum fluorescence (right) (ipsiversive, n = 12 neurons, contraversive, n = 22 neurons, *p = 0.0104, unpaired t test). *p < 0.05. Data are presented as mean ± SEM.

See also Figure S2 and Table S1.

CellPress
OPEN ACCESS

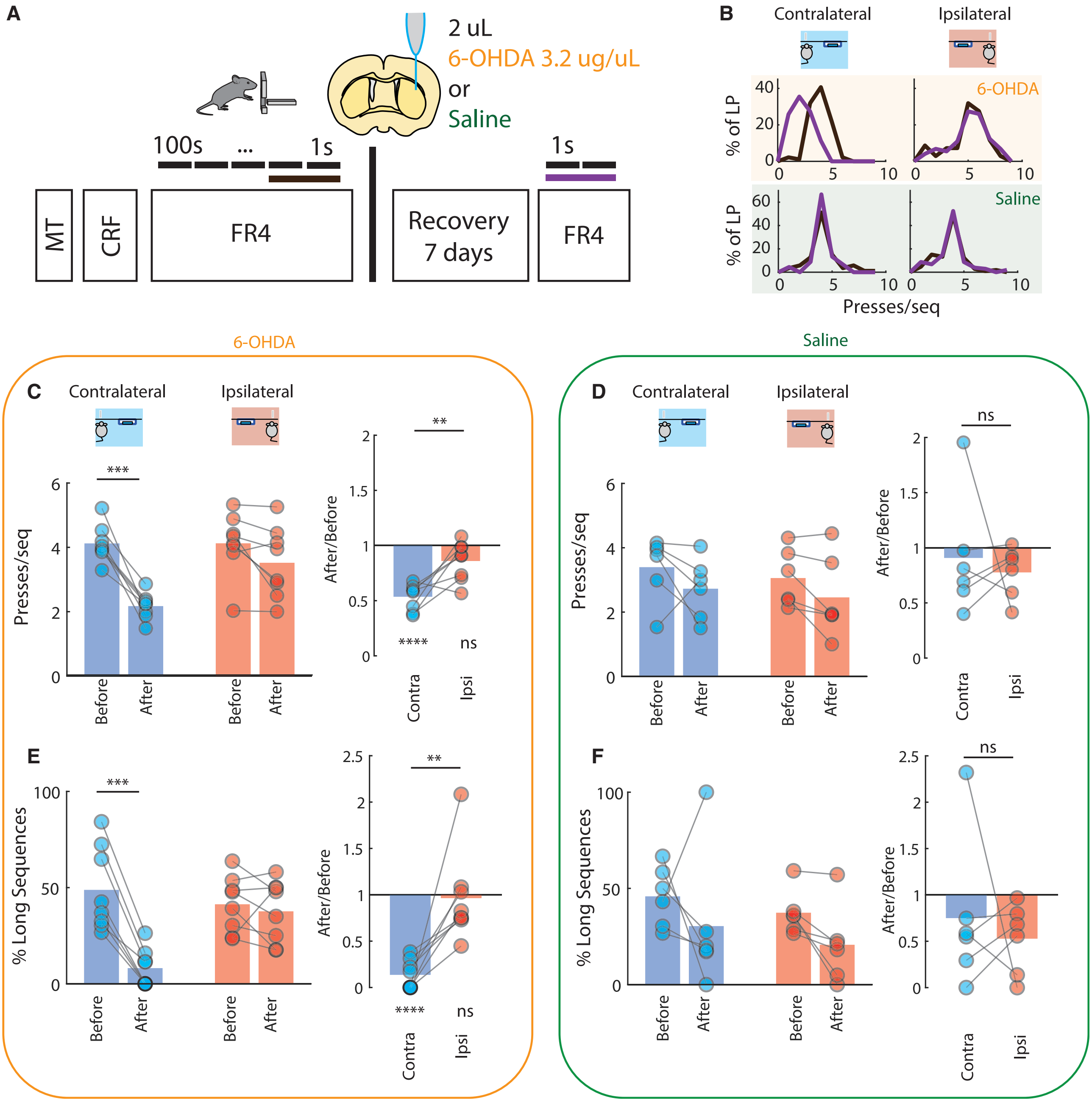

Figure 5. Dopamine depletion reduces the length of contralateral movement sequences

(A) A new group of mice (n = 14) was trained in the task, performing actions with each forepaw. After a plateau performance was reached, mice were injected with 6-OHDA or saline unilaterally in the striatum and retested after the lesion.

(B) Example of performance with contralateral and ipsilateral forelimbs to the lesion side, before (black) and after (purple) treatment of a mouse injected with 6-OHDA (top) and saline (bottom). Intra-striatal treatment with 6-OHDA leads to a redistribution of number of presses/sequence performed on the contralateral forelimb with the performance of sequences.

(C) Change in the number of presses/sequence for 6-OHDA-treated animals. (Left) Number of presses/sequence across the four conditions (time: before/after, and forelimb: contra/ipsilateral) for 6-OHDA-treated animals. There was an effect for time and an interaction between forelimb and time conditions. Two-way repeated-measures ANOVA, time $F(1,7) = 68.90$, $p < 0.001$, forelimb $F(1,7) = 4.704$, $p = 0.0667$, time x forelimb $F(1,7) = 11.11$, $p = 0.0125$. Post hoc tests revealed a significant difference in the before/after condition in the contralateral forelimb (4.12 ± 0.20 to 2.17 ± 0,14, multiple comparison test after two-way repeated-measures ANOVA, $t(7) = 6.835$, $p < 0.001$) but not ipsilaterally (4.13 ± 0.34 to 3.52 ± 0.39, multiple comparison test after two-way repeated-measures ANOVA, $t(7) = 2.121$, $p = 0.1380$). (Right) Ratio of presses/sequence after treatment, normalized to the one before treatment for both ipsilateral and contralateral forelimbs (normalization of data presented on the left). There was a significant difference between ipsilateral and contralateral change (paired t test, $t(7) = 3.759$, $p = 0.0071$)

(legend continued on next page)

However, this signal was only related to the length of contralateral (but not ipsilateral) sequences. Also, striatal DA depletion disrupted the length of contralateral, but not ipsilateral, movement sequences. By contrast, responses of reward-modulated SNc neurons were bilateral and modulated irrespective of the side of the preceding action. These results uncover a previously unknown relationship between the activity of DANs before movement and the length of movements.

Laterality is a major topic in nervous system organization, and most attention on the nigro-striatal pathway has been placed on dopaminergic striatal terminals. Although in freely moving animals striatal DA transients are synchronized across hemispheres,[30] contralateral action response was identified in DA terminals in the dorsal striatum.[7] SNc neurons have both ipsilateral and contralateral functionally relevant projections,[31] but those ipsilateral to SNc cell bodies are anatomically overrepresented.[32] Our data suggest that activity in DAN cell bodies is lateralized, and hence the higher activity in striatal DA terminals observed before contralateral movements is likely driven by increased firing rate and not enhanced terminal modulation of DA release. Furthermore, and given that DA depletion starts asymmetrically by the caudal putamen in PD,[19,33] these findings have implications for understanding the asymmetry in movement vigor observed in PD.[12,34]

It has been previously shown that DA and its metabolite 3,4-dihydroxyphenylacetic acid (DOPAC) increase bilaterally in the striatum in relation to speed when rodents run straight, but contralaterally when they perform circling movements.[35] This is in line with the asymmetry in SNc neuronal activation we observed, and suggests that asymmetric tonic levels of DA in the dorsal striatum regulate movement vigor.[36] 6-OHDA impairs both phasic and tonic DA activity. However, our results suggest that rapid changes in SNc DA preceding movement onset, in addition to tonic activity, do not modulate the general motivation to move. The observed lateralized response would be better explained considering a model of lateralized motivation signaling, or lateralized modulation of motor aspects.

Although evidence of laterality is present in movement responses, the same is not true for reward responses. This activity was similar, irrespective of the side of the performed action that led to it, and started only after reward collection. The lack of laterality of reward responses was also noted in a fiber-photometry study in the ventral striatum, where headfixed mice had to perform an ipsilateral or contralateral movement in response to a visual stimulus.[37] This suggests that responses to unpredicted rewards represent a more general teaching signal in the brain and are not solely related to the action performed to obtain the reward.

Activity in dorsal striatum DA terminals has been assessed during decision-making tasks.[37,38] In these tasks, evidence of lateralized DA responses is present when unilateral ambiguous sensory cues are presented[37] or when a choice is reported by the animal with a lateralized movement.[37,38] Although efforts have been made to unify observations of movement and reward responses in DANs, under classic reward prediction error (RPE) model, previous evidence suggest that movement signals may be modulated distinctly from RPE.[37,38] The results presented here also suggest that movement signals and reward-modulated signals can be independently modulated. If movement-modulated signals reflected learned action value, we would not expect a difference in magnitude of activity based on which limb was used to perform the actions, as reward prediction is similar for both left and right limb performance of action sequences. Furthermore, we would not expect that magazine-approach activity[28] would be higher for contraversive than for ipsiversive movements as, again, these actions have the same expected value. These observations do not argue that DANs do not encode an RPE, just that not all DANs necessarily encode an RPE. Facing the high dimensionality of an organism's behavior, error signals could be differently decomposed according to the condition[39] or different state information may be carried by

N = 8. Although contralaterally the change was significantly different from the unit value (one sample t test vs. 1: $t(7) = 11.07$, $p < 0.0001$), ipsilaterally no significant difference was found (one sample t test vs. 1: $t(7) = 2.281$, $p = 0.0565$).

(D) Change in the number of presses/sequence for saline-treated animals. (Left) Number of presses/sequence across the 4 conditions (time: before/after and forelimb: contra/ipsilateral) for saline-treated animals. There was only small effect for time. Two-way repeated-measures ANOVA, time: $F(1,5) = 7.704$, $p = 0.0391$, forelimb: $F(1,5) = 2.007$, $p = 0.2157$, time x forelimb: $F(1,5) = 0.01041$, $p = 0.9227$. There was not any significant change in the number of presses/sequence after saline treatment, either contralaterally (3.40 ± 0.35 to 2.73 ± 0.33, multiple comparison test after two-way repeated-measures ANOVA, $t(5) = 1.408$, $p = 0.3885$) or ipsilaterally (3.06 ± 0.31 to 2.46 ± 0.45, multiple comparison test after two-way repeated-measures ANOVA, $t(5) = 1.264$, $p = 0.4552$). (Right) Ratio of presses/sequence after treatment, normalized to the one before treatment for both ipsilateral and contralateral forelimbs (normalization of data presented on the left panel). There was no significant difference between ipsilateral and contralateral change (paired t test, $t(5) = 0.4441$, $p = 0.6755$).

(E) Change in the percentage of long sequences/all sequences for 6-OHDA-treated animals. Long sequences are defined as sequences with a number of presses higher than the mean number of presses in baseline condition for each forelimb (i.e., the number calculated in C and D). (Left) Percentage of long sequences/all sequence across the 4 conditions (time: before/after and forelimb: contra/ipsilateral) for 6-OHDA-treated animals. There was an effect for time and an interaction between forelimb and time conditions. Two-way repeated-measures ANOVA, time $F(1,7) = 30.12$, $p < 0.001$, forelimb $F(1,7) = 2.087$, $p = 0.1918$, time × forelimb $F(1,7) = 32,45$, $p < 0.001$. Post hoc tests revealed a significant difference in the before/after condition in the contralateral forelimb (48.82% ± 7.72% to 8.13% ± 3.48%, $t(7) = 8.854$, $p < 0.001$) but not in ipsilateral forelimbs (41.34% ± 5.16% to 37.67% ± 5.67%, $t(7) = 0.799$, $p = 0.9725$). (Right) Ratio of long sequences after treatment, normalized to the one before treatment for ipsilateral and contralateral forelimbs. There was a significant difference between ipsilateral and contralateral change (paired t test, $t(7) = 4.126$, $p = 0.0044$). Although contralaterally the change was significantly different from the unit value (one sample t test vs. 1: $t(7) = 15.46$, $p < 0.0001$), ipsilaterally no significant difference was found (one sample t test vs. 1: $t(7) = 0.210$, $p = 0.8397$).

(F) Change in the percentage of long sequences/all sequences for saline-treated animals. (Left) Percentage of long sequences/all sequence across the 4 conditions (time: before/after and forelimb: contra/ipsilateral) for saline-treated animals. There were no significant changes with saline treatment (two-way repeated-measures ANOVA, time $F(1,5) = 4.911$, $p = 0.078$, side $F(1,5) = 0.8281$, $p = 0.405$, time × side $F(1,5) = 0.003$, $p = 0.957$; contralateral: 45.87% ± 7.11% to 30.40% ± 14.40; ipsilateral: 37.35% ± 4.72% to 20.69% ± 8.20%). (Right) Ratio of long sequences after treatment, normalized to the one before treatment for ipsilateral and contralateral forelimbs. There was no significant difference between ipsilateral and contralateral change (paired t test, $t(5) = 0.5099$, $p = 0.6319$).

Data are presented as mean ± SEM. *$p < 0.05$; **$p < 0.01$; ***$p < 0.001$; ****$p < 0.0001$.

See also Figures S4 and S5, Table S1, and Videos S3 and S4.

dissociable parallel circuits.[40,41] However, this substantiates the existence of a population of SNc DANs directly involved in movement and movement invigoration—relevant for the interpretation of symptoms in scenarios of DA depletion. Chronic DA depletion is associated with vigor impairment,[15] paired with a lower spiny projection neurons (SPNs) recruitment. DA is known to modulate SPNs activity,[25] and evidence points toward more vigorous movements being related to larger recruitment of SPNs than less vigorous movements.[42] With a fast lever-pressing task, it was shown that the striatum represents sequences as single actions and that there are SPNs that exhibit sustained activity during the whole sequence performance. Importantly, the activity of these cells is positively correlated with the lever-press frequency and these SPNs are mostly D1 neurons.[43] In our view, action plans and context converge to the striatum through cortical and thalamic inputs, and fast changes in the concentration of DA in the striatum could modulate the variability in the activation of SPNs.[44] Thus, it is plausible that an increase in DA release in the striatum before sequence initiation would favor the excitability of these D1-SPNs, which in our task could translate into an increase in the number of lever presses.

Studies assessing DA terminals' activity in the striatum have consistently found that activity in the ventral striatum[1,7,37] has been related to reward and does not seem to be lateralized, while activity in the dorsal striatum is related to movement[1,38] and movement vigor[1] with lateralized responses.[37,38] These studies have used fiber-photometry techniques, where a full signal results from a composition of different components. Besides our study, other single-cell-resolution studies have demonstrated that different signals are segregated in DANs,[2,45] with neurons not only responding to reward but also encoding a range of behavioral variables, such as kinematics, cues, and performance accuracy. The findings here deserve further expansion regarding some aspects of PD etiopathogenesis. First, the involvement of distinct SNc populations in movement initiation vs. reward (an observation already described at axonal terminals in the striatum[1]) is in keeping with the notion of selective vulnerability of nigrostriatal degeneration and the origin of motor vs. neuropsychiatric manifestations such as depression, anxiety, or apathy.[46] Second, the finding that SNc activity is directly linked to the execution and vigor of a learned movement sequence implicates, contrary to classic understanding,[47] that the nigrostriatal dopaminergic system may continue to be activated and engaged during the performance of routine, automatic actions. Such neurons in the ventrolateral SNc are the first and most affected in PD and, accordingly, the findings here agree with the hypothesis of high metabolic demand and overuse as critical vulnerability factors underlying the onset of neurodegeneration.[48] Thus, clarifying whether there is a link between genetic heterogeneity and functional phenotypes in these dopaminergic neurons could provide valuable resources to better understand the spectrum of clinical manifestations in PD and, more importantly, to define the origin of selective neuronal dopaminergic degeneration in PD.

STAR★METHODS

Detailed methods are provided in the online version of this paper and include the following:

- KEY RESOURCES TABLE
- RESOURCE AVAILABILITY
  - Lead contact
  - Materials availability
  - Data and code availability
- EXPERIMENTAL MODEL AND SUBJECT DETAILS
  - Virus injections and lens placement
  - Single-limb fast FR4 operant task
  - GCaMP6f imaging using a mini-epifluorescence microscope
  - Calcium image processing and analysis
  - Cell pairing across sessions
  - PETH correlation across sessions
  - Sequence length analysis
  - Striatal injection of 6-OHDA or Saline and post-operative care
  - Sequence length analysis
  - Reanalysis of optogenetic experimental data
  - Anatomical verification
- QUANTIFICATION AND STATISTICAL ANALYSIS

SUPPLEMENTAL INFORMATION

Supplemental information can be found online at https://doi.org/10.1016/j.cub.2024.01.067.

ACKNOWLEDGMENTS

We thank Ana Vaz and Catarina Carvalho for mouse colony management, Thomas Akam and Hélio Rodrigues for help in behavioral box development and implementation, and the Champalimaud Hardware Platform (Filipe Carvalho, Artur Silva, and Dário Bento) for support in the development of the behavioral hardware setup. We thank Cristina Alcácer and Nuno Loureiro for their contributions during the 6-OHDA experiments. This work was supported by Fundação Para a Ciência e Tecnologia (FCT) through a doctoral fellowship (SFRH/BD/119623/2016 to M.D.M.) and Fundação Luso-Americana para o Desenvolvimento (FLAD) by a visiting student fellowship (2018/31 to M.D.M.); by a doctoral fellowship from the Gulbenkian Foundation (to J.A.d.S.), a Marie Curie Fellowship (MSCA-IF-RI 2016 to L.F.H.), and a Spanish Ministry of Innovation and Sciences doctoral fellowship (BES-2016-077493 to I.C.); and by ERA-NET, ERC (COG 617142), HHMI (IEC 55007415), National Institutes of Health (5U19NS104649), and the Simons-Emory International Consortium on Motor Control and the Aligning Science Across Parkinson's (ASAP-020551) through the Michael J. Fox Foundation for Parkinson's Research (MJFF) to R.M.C. Further support was obtained from the research infrastructure Congento, co-funded by Lisboa2020 and FCT (LISBOA-01-0145-FEDER-022170).

AUTHOR CONTRIBUTIONS

M.D.M., J.A.d.S., and R.M.C. designed experiments and conceptualized analysis. M.D.M., J.A.d.S., and L.H. performed behavioral experiments. M.D.M., L.H., and I.C. performed calcium imaging experiments. M.D.M. performed analysis, with contribution from J.A.d.S. M.D.M. wrote the original draft with J.A.d.S. and R.M.C., and it was critically reviewed by the other authors. R.M.C. and J.O. supervised the work.

DECLARATION OF INTERESTS

The authors declare no competing interests.

Received: February 13, 2022
Revised: December 1, 2023
Accepted: January 26, 2024
Published: February 19, 2024

REFERENCES

1. Howe, M.W., and Dombeck, D.A. (2016). Rapid signalling in distinct dopaminergic axons during locomotion and reward. Nature *535*, 505–510.
2. da Silva, J.A., Tecuapetla, F., Paixão, V., and Costa, R.M. (2018). Dopamine neuron activity before action initiation gates and invigorates future movements. Nature *554*, 244–248.
3. Ehringer, H., and Hornykiewicz, O. (1960). Distribution of noradrenaline and dopamine (3-hydroxytyramine) in the human brain and their behavior in diseases of the extrapyramidal system. Klin. Wochenschr. *38*, 1236–1239.
4. Schultz, W., Ruffieux, A., and Aebischer, P. (1983). The activity of pars compacta neurons of the monkey substantia nigra in relation to motor activation. Exp. Brain Res. *51*, 377–387.
5. Romo, R., and Schultz, W. (1990). Dopamine neurons of the monkey midbrain: contingencies of responses to active touch during self-initiated arm movements. J. Neurophysiol. *63*, 592–606.
6. Jin, X., and Costa, R.M. (2010). Start/stop signals emerge in nigrostriatal circuits during sequence learning. Nature *466*, 457–462.
7. Parker, N.F., Cameron, C.M., Taliaferro, J.P., Lee, J., Choi, J.Y., Davidson, T.J., Daw, N.D., and Witten, I.B. (2016). Reward and choice encoding in terminals of midbrain dopamine neurons depends on striatal target. Nat. Neurosci. *19*, 845–854.
8. Collins, A.L., Greenfield, V.Y., Bye, J.K., Linker, K.E., Wang, A.S., and Wassum, K.M. (2016). Dynamic mesolimbic dopamine signaling during action sequence learning and expectation violation. Sci. Rep. *6*, 20231.
9. Dodson, P.D., Dreyer, J.K., Jennings, K.A., Syed, E.C., Wade-Martins, R., Cragg, S.J., Bolam, J.P., and Magill, P.J. (2016). Representation of spontaneous movement by dopaminergic neurons is cell-type selective and disrupted in parkinsonism. Proc. Natl. Acad. Sci. USA *113*, E2180–E2188.
10. Hallett, M., and Khoshbin, S. (1980). A physiological mechanism of bradykinesia. Brain *103*, 301–314.
11. Bologna, M., Leodori, G., Stirpe, P., Paparella, G., Colella, D., Belvisi, D., Fasano, A., Fabbrini, G., and Berardelli, A. (2016). Bradykinesia in early and advanced Parkinson's disease. J. Neurol. Sci. *369*, 286–291.
12. Mazzoni, P., Hristova, A., and Krakauer, J.W. (2007). Why don't we move faster? Parkinson's disease, movement vigor, and implicit motivation. J. Neurosci. *27*, 7105–7116.
13. Dowd, E., and Dunnett, S.B. (2005). Comparison of 6-hydroxydopamine-induced medial forebrain bundle and nigrostriatal terminal lesions in a lateralised nose-poking task in rats. Behav. Brain Res. *159*, 153–161.
14. Palmiter, R.D. (2008). Dopamine signaling in the dorsal striatum is essential for motivated behaviors: lessons from dopamine-deficient mice. Ann. N. Y. Acad. Sci. *1129*, 35–46.
15. Panigrahi, B., Martin, K.A., Li, Y., Graves, A.R., Vollmer, A., Olson, L., Mensh, B.D., Karpova, A.Y., and Dudman, J.T. (2015). Dopamine Is Required for the Neural Representation and Control of Movement Vigor. Cell *162*, 1418–1430.
16. Shah, V.V., McNames, J., Mancini, M., Carlson-Kuhta, P., Spain, R.I., Nutt, J.G., El-Gohary, M., Curtze, C., and Horak, F.B. (2020). Quantity and quality of gait and turning in people with multiple sclerosis, Parkinson's disease and matched controls during daily living. J. Neurol. *267*, 1188–1196.
17. Niv, Y., Daw, N.D., Joel, D., and Dayan, P. (2007). Tonic dopamine: opportunity costs and the control of response vigor. Psychopharmacol. (Berl.) *191*, 507–520.
18. Berke, J.D. (2018). What does dopamine mean? Nat. Neurosci. *21*, 787–793.
19. Monje, M.H.G., Sánchez-Ferro, Á., Pineda-Pardo, J.A., Vela-Desojo, L., Alonso-Frech, F., and Obeso, J.A. (2021). Motor Onset Topography and Progression in Parkinson's Disease: the Upper Limb Is First. Mov. Disord. *36*, 905–915.
20. Roggendorf, J., Chen, S., Baudrexel, S., van de Loo, S., Seifried, C., and Hilker, R. (2012). Arm swing asymmetry in Parkinson's disease measured with ultrasound based motion analysis during treadmill gait. Gait Posture *35*, 116–120.
21. Carli, M., Evenden, J.L., and Robbins, T.W. (1985). Depletion of unilateral striatal dopamine impairs initiation of contralateral actions and not sensory attention. Nature *313*, 679–682.
22. Schwarcz, R., Fuxe, K., Agnati, L.F., Hökfelt, T., and Coyle, J.T. (1979). Rotational behavior in rats with unilateral striatal kainic acid lesions: a behavioural model for studies on intact dopamine receptors. Brain Res. *170*, 485–495.
23. Kitama, T., Ohno, T., Tanaka, M., Tsubokawa, H., and Yoshida, K. (1991). Stimulation of the caudate nucleus induces contraversive saccadic eye movements as well as head turning in the cat. Neurosci. Res. *12*, 287–292.
24. Tecuapetla, F., Matias, S., Dugue, G.P., Mainen, Z.F., and Costa, R.M. (2014). Balanced activity in basal ganglia projection pathways is critical for contraversive movements. Nat. Commun. *5*, 4315.
25. Lahiri, A.K., and Bevan, M.D. (2020). Dopaminergic Transmission Rapidly and Persistently Enhances Excitability of D1 Receptor-Expressing Striatal Projection Neurons. Neuron *106*, 277–290.e6.
26. Ghosh, K.K., Burns, L.D., Cocker, E.D., Nimmerjahn, A., Ziv, Y., Gamal, A.E., and Schnitzer, M.J. (2011). Miniaturized integration of a fluorescence microscope. Nat. Methods *8*, 871–878.
27. Zhou, P., Resendez, S.L., Rodriguez-Romaguera, J., Jimenez, J.C., Neufeld, S.Q., Giovannucci, A., Friedrich, J., Pnevmatikakis, E.A., Stuber, G.D., Hen, R., et al. (2018). Efficient and accurate extraction of in vivo calcium signals from microendoscopic video data. eLife *7*, e28728.
28. Howe, M.W., Tierney, P.L., Sandberg, S.G., Phillips, P.E., and Graybiel, A.M. (2013). Prolonged dopamine signalling in striatum signals proximity and value of distant rewards. Nature *500*, 575–579.
29. Stott, S.R.W., and Barker, R.A. (2014). Time course of dopamine neuron loss and glial response in the 6-OHDA striatal mouse model of Parkinson's disease. Eur. J. Neurosci. *39*, 1042–1056.
30. Fox, M.E., Mikhailova, M.A., Bass, C.E., Takmakov, P., Gainetdinov, R.R., Budygin, E.A., and Wightman, R.M. (2016). Cross-hemispheric dopamine projections have functional significance. Proc. Natl. Acad. Sci. USA *113*, 6985–6990.
31. Jaeger, C.B., Joh, T.H., and Reis, D.J. (1983). The effect of forebrain lesions in the neonatal rat: survival of midbrain dopaminergic neurons and the crossed nigrostriatal projection. J. Comp. Neurol. *218*, 74–90.
32. Poulin, J.F., Caronia, G., Hofer, C., Cui, Q., Helm, B., Ramakrishnan, C., Chan, C.S., Dombeck, D.A., Deisseroth, K., and Awatramani, R. (2018). Mapping projections of molecularly defined dopamine neuron subtypes using intersectional genetic approaches. Nat. Neurosci. *21*, 1260–1271.
33. Morrish, P.K., Sawle, G.V., and Brooks, D.J. (1996). Regional changes in [18F]dopa metabolism in the striatum in Parkinson's disease. Brain *119*, 2097–2103.
34. Djaldetti, R., Ziv, I., and Melamed, E. (2006). The mystery of motor asymmetry in Parkinson's disease. Lancet Neurol. *5*, 796–802.
35. Freed, C.R., and Yamamoto, B.K. (1985). Regional brain dopamine metabolism: a marker for the speed, direction, and posture of moving animals. Science *229*, 62–65.
36. Schultz, W. (2002). Getting formal with dopamine and reward. Neuron *36*, 241–263.
37. Moss, M.M., Zatka-Haas, P., Harris, K.D., Carandini, M., and Lak, A. (2021). Dopamine Axons in Dorsal Striatum Encode Contralateral Visual Stimuli and Choices. J. Neurosci. *41*, 7197–7205.
38. Lee, R.S., Mattar, M.G., Parker, N.F., Witten, I.B., and Daw, N.D. (2019). Reward prediction error does not explain movement selectivity in DMS-projecting dopamine neurons. eLife *8*, e42992.
39. Diuk, C., Tsai, K., Wallis, J., Botvinick, M., and Niv, Y. (2013). Hierarchical learning induces two simultaneous, but separable, prediction errors in human basal ganglia. J. Neurosci. *33*, 5797–5805.
40. Takahashi, Y.K., Langdon, A.J., Niv, Y., and Schoenbaum, G. (2016). Temporal Specificity of Reward Prediction Errors Signaled by Putative

Dopamine Neurons in Rat VTA Depends on Ventral Striatum. Neuron *91*, 182–193.

41. Lau, B., Monteiro, T., and Paton, J.J. (2017). The many worlds hypothesis of dopamine prediction error: implications of a parallel circuit architecture in the basal ganglia. Curr. Opin. Neurobiol. *46*, 241–247.
42. Barbera, G., Liang, B., Zhang, L., Gerfen, C.R., Culurciello, E., Chen, R., Li, Y., and Lin, D.T. (2016). Spatially Compact Neural Clusters in the Dorsal Striatum Encode Locomotion Relevant Information. Neuron *92*, 202–213.
43. Jin, X., Tecuapetla, F., and Costa, R.M. (2014). Basal ganglia subcircuits distinctively encode the parsing and concatenation of action sequences. Nat. Neurosci. *17*, 423–430.
44. Klaus, A., Alves da Silva, J., and Costa, R.M. (2019). What, If, and When to Move: Basal Ganglia Circuits and Self-Paced Action Initiation. Annu. Rev. Neurosci. *42*, 459–483.
45. Engelhard, B., Finkelstein, J., Cox, J., Fleming, W., Jang, H.J., Ornelas, S., Koay, S.A., Thiberge, S.Y., Daw, N.D., Tank, D.W., et al. (2019). Specialized coding of sensory, motor and cognitive variables in VTA dopamine neurons. Nature *570*, 509–513.
46. Weintraub, D., Simuni, T., Caspell-Garcia, C., Coffey, C., Lasch, S., Siderowf, A., Aarsland, D., Barone, P., Burn, D., Chahine, L.M., et al. (2015). Cognitive performance and neuropsychiatric symptoms in early, untreated Parkinson's disease. Mov. Disord. *30*, 919–927.
47. DeLong, M.R., Crutcher, M.D., and Georgopoulos, A.P. (1983). Relations between movement and single cell discharge in the substantia nigra of the behaving monkey. J. Neurosci. *3*, 1599–1606.
48. Hernandez, L.F., Obeso, I., Costa, R.M., Redgrave, P., and Obeso, J.A. (2019). Dopaminergic Vulnerability in Parkinson Disease: The Cost of Humans' Habitual Performance. Trends Neurosci. *42*, 375–383.
49. Klaus A., Martins G.J., Paixao V.B., Zhou P., Paninski L., Costa R.M. (2017). The Spatiotemporal Organization of the Striatum Encodes Action Space. Neuron *95*, 1171–1180.e7.
50. Pnevmatikakis, E.A., Soudry, D., Gao, Y., Machado, T.A., Merel, J., Pfau, D., Reardon, T., Mu, Y., Lacefield, C., Yang, W., Ahrens, M., Bruno, R., Jessell, T.M., Peterka, D.S., Yuste, R., and Paninski, L. (2016). Simultaneous Denoising, Deconvolution, and Demixing of Calcium Imaging Data. Neuron *89*, 285–299.
51. Lopes, G., Bonacchi, N., Frazão, J., Neto, J.P., Atallah, B.V., Soares, S., Moreira, L., Matias, S., Itskov, P.M., Correia, P.A., et al. (2015). Bonsai: an event-based framework for processing and controlling data streams. Front Neuroinform. *9*, 7.

STAR★METHODS

KEY RESOURCES TABLE

REAGENT or RESOURCE	SOURCE	IDENTIFIER
Antibodies		
Anti-Tyrosine Hydroxylase antibody	Abcam	RRID: AB_112
Anti-Rabbit IgG H&L (Alexa Fluor® 555)	Abcam	RRID: AB_150078
Bacterial and virus strains		
AAV5.SYN.FlexGCaMP6fWPRE.SV40	UPENN	AV-5-PV2822, Addgene ID: 100837-AAV5
Chemicals, peptides, and recombinant proteins		
Sucrose	Sigma-Aldrich	Cat# 84099
6-Hydroxydopamine hydrobromide	Sigma-Aldrich	Cat# H116
Experimental models: Organisms/strains		
Mouse: B6.SJL-*Slc6a3*$^{tm1.1(cre)Bkmn}$/J (DAT-Cre)	Jackson Laboratories	RRID:IMSR_JAX:006660
Mouse: C57BL6	Champalimaud Centre Vivarium	N/A
Software and algorithms		
CNMF-e	Klaus et al.[49]; Pnevmatikakis et al.[50]	https://github.com/zhoupc/CNMF_E
Bonsai-Open Ephys	Lopes et al.[51]	RRID:SCR_021512; https://open-ephys.org/bonsai
pyControl	pyControl developers	RRID:SCR_021612; https://pycontrol.readthedocs.io
Matlab 2016b, Matlab 2018b	MathWorks	RRID:SCR_001622; https://www.mathworks.com/products/matlab.html
GraphPad Prism 8	GraphPad Software Inc.	RRID:SCR_002798; http://www.graphpad.com/
Mosaic Software v. 1.2.0	Inscopix	RRID:SCR_017408; https://support.inscopix.com/search/site/Mosaic
Zenodo: Data and code		https://doi.org/10.5281/zenodo.10493365

RESOURCE AVAILABILITY

Lead contact
Further information and requests for resources and reagents should be directed to and will be fulfilled by the lead contact, Rui M. Costa (rui.costa@alleninstitute.org).

Materials availability
This study did not generate new unique reagents.

Data and code availability
Source data from this study are available at https://doi.org/10.5281/zenodo.10493365. Any additional information required to reanalyze the data reported in this paper is available from the lead contact upon request.

EXPERIMENTAL MODEL AND SUBJECT DETAILS

All experiments were approved by the Portuguese Direcção Geral de Veterinária and Champalimaud Centre for the Unknown Ethical Committee and performed in accordance with European Union Directive for Protection of Vertebrates Used for Experimental and other Scientific Ends (86/609/CEE and Law No. 0421/000/000/2014). Male C57BL/6J mice were tested between 2 and 4 months old. For calcium imaging studies, the male DAT-IRES:Cre (Dopamine Transporter-Internal Ribosome Entry Site-linked Cre recombinase gene) mouse line from Jackson Labs Stock 006660 (The Jackson Laboratory; B6.SJL-Slc6a3tm1.1(Cre)Bkmn/J) was used. These mice have Cre recombinase expression directed to dopaminergic neurons, without disrupting endogenous dopamine

CellPress
OPEN ACCESS

transporter expression. These studies were only performed in male mice, thus limiting generalization to female animals. Genotype was confirmed by polymerase chain reaction (PCR) amplification. Sample sizes are detailed in the results and/or figure legends.

Virus injections and lens placement

Mice were kept in deep anaesthesia using a mixture of isoflurane and oxygen (1-3% isoflurane at 1l/min) and the procedure was conducted in aseptic conditions.

The mouse head was stabilized in the stereotaxic apparatus (Koft), a skin incision was performed to expose the skull, connective and muscle tissue was carefully removed and the skull surface was leveled at less than 0.05mm by comparing the height of bregma and lambda, and also in medial-lateral directions. Unilateral virus injection was performed using a glass pipette with GCaMP6f stock viral solution (AAV2/5.SYN.FlexGCaMP6fWPRE.SV40 - University of Pennsylvania). For imaging, 1 ul of virus solution was injected in the right (n=3) of the left (n=3) substantia nigra compacta at the following coordinates: -3.16 mm anteroposterior, 1.40mm lateral from bregma and 4.20 deep from the brain surface. The injection was done using a Nanojet II or Nanojet III (Drummond Scientific) with a rate of injection of 4.6 nl every 5s. After the injection was finished, the pipette was left in place for 10-15 minutes. The virus solution was kept at -80 ºC and thawed at room temperature just before the injection.

A 500-um diameter, 8.2-mm long gradient index (GRIN) lens (GLP-0584, Inscopix) was implanted at the same coordinates as the injection. Before the lens was lowered, a blunt 28 G needle was lowered to 3 mm deep from the brain surface to facilitate the lowering of the GRIN lens. The GRIN lens was then lowered (4.2 mm deep). The lens was fixed in place using cyanoacrilate, quick adhesive cement (C&B Metabond) and black dental cement (Ortho-Jet). Three weeks after surgery, the mouse was anaesthetized and fixed with head bars. A baseplante (BPC-2, Inscopix) attached to a mini epifluorescence microscope (nVista HD, Inscopix) was positioned above the GRIN lens. To correctly position the baseplate, brain tissue was imaged through the lens to find the appropriate focal plane using 40% LED power, a frame rate of 10 Hz and a digital gain of 4. Once the focal plane was set, the baseplate was cemented to the rest of the cap using the same dental cement. Imaging started 2–3 days after this final step. Detailed protocol can be found at: https://dx.doi.org/10.17504/protocols.io.81wgbx813lpk/v1

Single-limb fast FR4 operant task

Animals were trained using 14x16 cm custom-built operant chambers placed inside sound attenuating boxes. PyControl, (https://pycontrol.readthedocs.io), a behavioral experiment control system built around the Micropython microcontroller, was used to control and detect events and supply rewards. The custom-built boxes had in their design a retractable lever.

At the beginning of each session there was the onset of a light, and the animals were required to perform a sequence of presses at a minimum frequency in order to obtain a sucrose reward. Sucrose solution (10%) was delivered through the opening of a solenoid (LHDA1231515H, Lee Company). Sucrose solution was delivered through a tube into the magazine (5μl per reward). Licks were detected using an infrared beam and through a side camera, mouse position in the box was monitored through a camera placed on the top of the box.

Mice were placed on food restriction throughout training, and fed daily after the training sessions with approximately 1.5 - 2.5g of regular food to allow them to maintain a body weight of around 85% of their baseline weight. To facilitate learning, animals were initially exposed to one session of magazine training where sucrose would be available on a random time schedule, and to three to four sessions of continuous reinforcement schedule (CRF) before training, where single lever presses would be reinforced. In the following sessions animals were reinforced if they performed a sequence of 4 consecutive presses (Fixed Ratio 4, FR4) in a particular time window (FR4/Xs, fixed-ration four within X seconds). The duration of time required to perform the four lever presses was reduced across sessions from 100 seconds to 20 s, 8 s, 4 s, 2 s and finally 1s. To shape animals to use only one of the forelimbs the lever was progressively retracted and the slit thought which the forelimb accessed the lever was reduced with a custom-built piece.

In the imaging group, animals performed the task 2 times/session - one with the lever in the left side of the box and the other with the lever in the right - that were randomized throughout the training. Task ended after 30 minutes on each side or when the animals obtained 30 rewards.

The lever was equipped with a digital 9-axis inertial sensor with a sampling rate of 200 Hz (MPU-9150, Invensense) assembled on a custom-made PCB and connected to a computer via a custom-made USB interface PCB (Champalimaud Foundation Hardware Platform). Lever velocity was extracted from this sensor.

Timestamps from the behavioral task were synchronized with calcium imaging data using TTL pulses sent from the behavioral chambers to the Inscopix data acquisition system via a BNC cable. Detailed protocol can be found at: https://dx.doi.org/10.17504/protocols.io.5qpvo3439v4o/v1

GCaMP6f imaging using a mini-epifluorescence microscope

Mice were briefly anaesthetized using a mixture of isoflurane and oxygen (1% isoflurane at 1L/min) and the mini-epifluorescence microscope was attached to the baseplate. This was followed by a period of 15-20 min of recovery in the home cage before starting the experiments. Fluorescence images were acquired at 10 Hz and the LED power was set 40-60% with a gain of 4. Image acquisition parameters were always set to the same parameters between sessions to be able to compare the activity recorded. Six GCaMP6f-expressing DAT-Cre mice were imaged during the FR4/1s task in 3 performance days. Detailed protocol can be found at: https://dx.doi.org/10.17504/protocols.io.kqdg3xdx7g25/v1

Calcium image processing and analysis

GCaMP6f image processing

All fluorescence movies were initially processed using the Mosaic Software (v. 1.2.0, Inscopix). Two different movies were collected on the same day (one for ipsi and one for contralateral forelimb). As the epifluorescence microscope was not removed during this period, movies were concatenated for the next analysis step. First, all frames were spatially binned by a factor of 4. To correct the movie for translational movements and rotations, frames were registered to a reference image consisting of an average of the raw fluorescence movie.

Extraction of calcium signals

We implemented the 'constrained non-negative matrix factorization for endoscopic data' (CNMF-E) framework for our calcium imaging analysis. This framework is an adaptation of the CNMF algorithm that can reliably deal with the large fluctuating background from multiple sources in the data, and enable accurate source extraction of cellular signals. It include four steps: 1) initialize spatial and temporal components of single neurons without the direct estimation of the background, 2) estimate the background given the estimated spatiotemporal activity of the neurons; 3) update the spatial and temporal components of all neurons while fixing the estimated background fluctuation, 4) iteratively repeat step 2 and 3.

CNMF-E only identifies regions of interest that are active in the condition.

After analysis, data from the videos were separated in ipsi and contralateral videos. Further calcium imaging analyses were performed on standardized scores (z-score) of each session.

Criteria to identify lever-press-related and reward-related DANs using GCaMP6f imaging

We constructed a PETH for each neuron trace spanning from -8 to 6 s from lever press onset for the first press and for the first lick after reward. Distributions of the PETH from -8 to -3 s before the event were considered baseline activity. We then searched each PETH during a determined epoch for bins that were significantly different from the baseline. A significant change in fluorescence was defined as at least two consecutive bins with fluorescence higher than a threshold of 99% above the baseline. For first-press modulated neurons, a window from -2 to 0 s was used. For rewarded lick modulated neurons a window from 0 to 1 s was used. For each neuron, maximum activity in specific time-windows was calculated by the maximum of a moving average of 3 bins.

Cell pairing across sessions

Analysis of matched cells between different days/sessions was based on a nearest neighbors' method. For all sessions, a centroid for each ROI was calculated. For each reference centroid, distance from all centroids on the image to be compared was calculated, and the 3 ROIs with the smallest distance were visually inspected for their shape to define a match. Alignments were performed to 4 events: First lever press and Reward in the ipsi and contralateral situations. The calcium-signal from -10 to +6 seconds after the event was used to calculate the correlation coefficients for individual ROIs across days in the 4 conditions. If the neuron was matched in the 3 days, the 3 possible correlation coefficients were calculated and averaged. Then the maximum value was extracted. As a control, we correlated each ROI with all ROIs in the field of view (FOV) of the same animal on the comparison day. The maximum value was extracted and then averaged across units. Correlation values were transformed for each point using Fisher's Z statistic, then the samples were averaged, and back-transformed into a weighted correlation.

PETH correlation across sessions

For each ROI, 4 PETHs were built as previously described: For the ipsilateral and contralateral conditions 2 events were considered (lever press and rewarded licks). For each ROI pair the Spearman correlation coefficient between these 4 events was calculated. The one with the maximum correlation was identified and this value extracted. This process was repeated when matching occurred across the 3 sessions, and average value between the 3 matchings (A and B, A and C and B and C) was computed. These are the values for matched ROIs in Figure 3D.

As control, we ran the same analysis, but instead of using the matched ROI we used all ROIs from the same animal, in a different session (different group). For the shuffled control we ran the same analysis but performed a time-shuffle of the matched ROIs from across different sessions (shuffled group). We then calculated the average of the maximum correlations as previously described.

Sequence length analysis

A movement sequence was defined as a bout of lever presses occurring while the animal's snout in spatially defined region of interest near the lever. A lever press can occur isolatedly – i.e., preceded and succeeded by the entrance / exit of the lever region – or in groups of presses. A bout of presses can have from 1 to any positive number of presses. For imaging experiments, as the cable could cause some distortion on head position estimate, we confirmed these results by defining a second ROI using the side camera. Sequences were divided by the number of presses performed. We grouped together sequences composed of 1 or 2 presses as well sequences with 7 or more presses. A neuron was classified as sequence length-modulated if the non-parametric correlation between its maximum activity and number of presses was significant ($p<0.05$).

Striatal injection of 6-OHDA or Saline and post-operative care

As in virus injections, mice were kept in deep anaesthesia using a mixture of isoflurane and oxygen (1-3% isoflurane at 1l/min) and the procedure was conducted in aseptic conditions.

CellPress
OPEN ACCESS

The mouse head was stabilized in the stereotaxic apparatus (Koft), a skin incision was performed to expose the skull, connective and muscle tissue were carefully removed and the skull surface was leveled at less than 0.05 mm by comparing the height of bregma and lambda, and also in medial-lateral directions. 6-Hydroxydopamine hydrochloride (Sigma Aldrich AB, Sweden) was dissolved at a fixed concentration of 3.2 μg/μl free-base in 0.02% ice-cold ascorbic acid/saline and used within 2 h. Injection of 2 ul of 6-OHDA or saline was performed in the dorsolateral striatum +0.5 mm anteroposterior and ±2.5 mm lateral from bregma and 3.0 mm deep. Injection was done through a glass pipette using a Nanojet II with a rate of injection of 4.6 nl every 5s. After the injection was finished, the pipette was left in place for 10-15 minutes.

After surgery, food restriction was reduced (with each animal having access to up 4 mg of food pellets/day). Mice that showed weight loss were hand-fed (i.e. they were presented with the food while being held by the hands of the investigator) and DietGel Boost was placed in their boxed up to 4 days after surgery. In order to avoid competition for the food, weaker mice were placed in cages other than those containing unimpaired mice. The postoperative survival rate was 100%. Detailed protocol can be found at: https://dx.doi.org/10.17504/protocols.io.x54v9p4pzg3e/v1

Sequence length analysis

Movement sequences were divided into short and long sequences according to mean number of presses/sequence before striatal injection of 6-OHDA or Saline.

Reanalysis of optogenetic experimental data

Data reported of Figure S4D is a new analysis of a dataset available from a previous publication (2). In summary, AAV2/1.CAG.Flex.ArchT-GFP (titre 1.4 × 1012, University of Pennsylvania) and AVV2/1.EF1a.DIO. eYFP (titre 1.4 × 1013, University of North Carolina) were injected bilaterally at 2.3 nl evey 5 s in the same coordinate previously described. Optical fibres with a diameter of 230 μm and a NA of 0.39 (Thorlabs FMT 200 EMT) were bilaterally placed at a depth of 3.9 from the brain surface in TH-Cre male mice from the FI12 mouse line. Light from free-launched 500-mW, 556-nm, diode-pumped, solid-state laser from CNI Lasers, controlled using an AOM (AA Optolectronic), was delivered after being captured by a collimator and split using a one-input to two-outputs rotary joint (Doric Lenses). Behaviour training and testing took place in operant chambers within a sound-attenuating box (Med-Associates) and equipped with one retractable lever on the left side of the food magazine and a house light (3 W, 24 V). Sucrose solution (10%) was delivered into a metal cup in the magazine through a syringe pump (20 μ l per reward). Magazine entries were recorded using an infrared beam and licks using a contact lickometer. Mice were placed on food restriction throughout training, and they performed a fixed ratio schedule in which eight presses earn a reinforcer (FR8), without any stimulus signaling when eight presses were completed. Two different light delivery schedules were used: continuous light delivery for 5 s before the first lever press in a sequence; and continuous light delivery for 5 s after the first lever press in a sequence. For the first condition, the triggering of the light was contingent on the breaking of an infrared beam (IRB) positioned right next to the magazine, on the side of the lever.

Anatomical verification

Animals were euthanized after completion of the behavioural tests. First animals were anaesthetized with isoflurane, followed by intraperitoneal injection of ketamine-xylazine (5 mg/kg xylazine; 100 mg/kg ketamine). Animals were then perfused with 1% phosphate buffered saline (PBS) and 4% paraformaldehyde and brains were extracted for histological processing. Brains were kept in 4% paraformaldehyde overnight and then transferred to 1x PBS solution. Brains were sectioned coronally in 50-um slices (using a Leica vibratome VT1000S) and kept in PBS before mounting or immunostaining.

Images were taken using a wide-field fluorescence microscope (Zeiss AxioImager).

Detailed protocol can be found at: https://dx.doi.org/10.17504/protocols.io.14egn3o3ql5d/v1

QUANTIFICATION AND STATISTICAL ANALYSIS

Data is presented as mean ± standard error of mean (SEM) and statistical significance was considered for $p < 0.05$. Statistical analysis was conducted using GraphPad Prism 8 (GraphPad Software Inc., CA) and MATLAB statistical toolbox (The MathWorks Inc, MA). One-way or two-way ANOVAs were used to investigate main effects, and Šídák-corrected post-hoc comparisons performed whenever appropriate. Paired or unpaired t-tests were used for planned comparisons. Parametric tests were used as distributions were normal. For Figure 3, Spearman's rank correlations were calculated. Details for statistical tests are presented in Table S1. Statistical methods were not used to pre-determine sample size.

Current Biology, Volume *34*

Supplemental Information

Dopamine neuron activity encodes the length of upcoming contralateral movement sequences

Marcelo D. Mendonça, Joaquim Alves da Silva, Ledia F. Hernandez, Ivan Castela, José Obeso, and Rui M. Costa

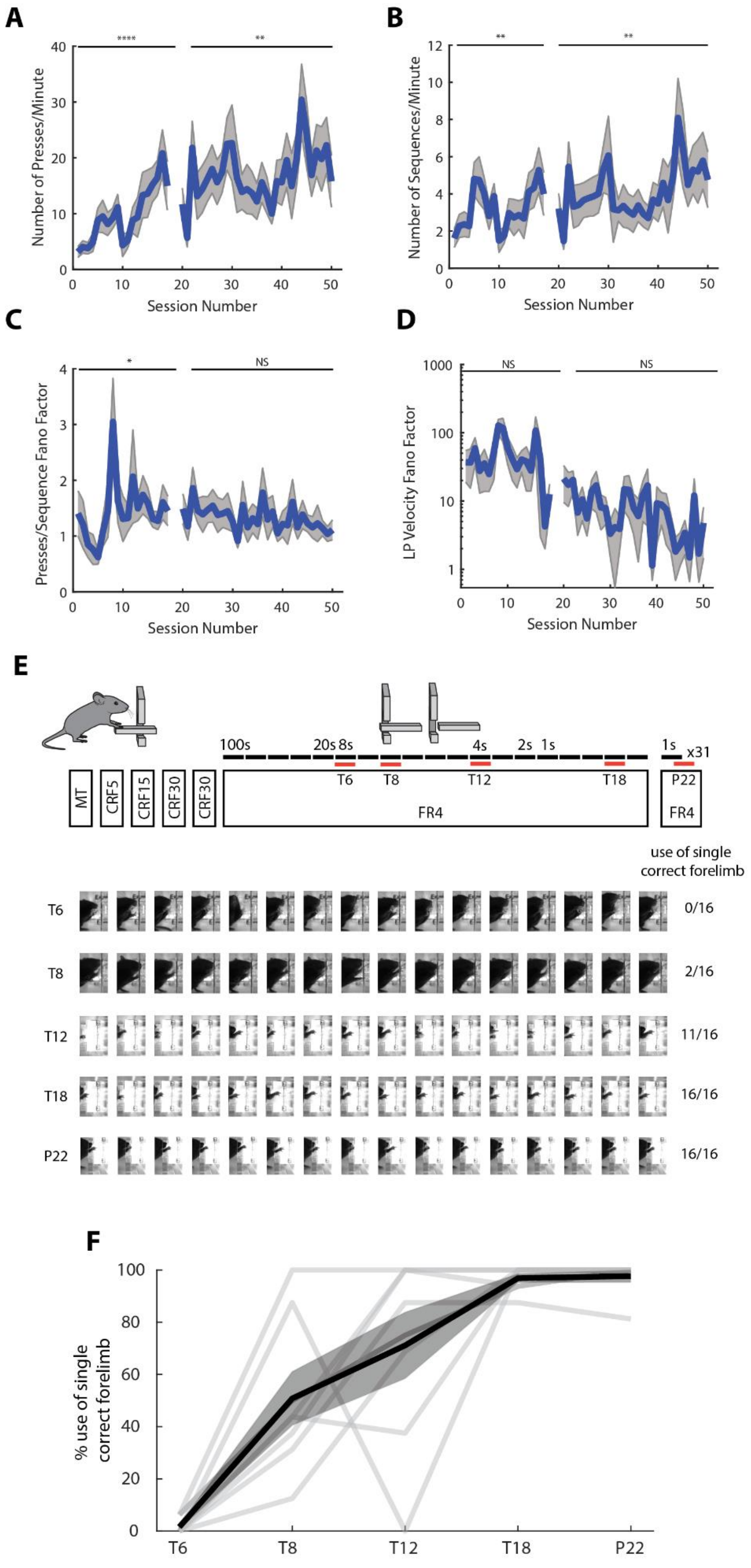
A
****
**
Number of Presses/Minute
Session Number
B
**
**
Number of Sequences/Minute
Session Number
C
*
NS
Presses/Sequence Fano Factor
Session Number
D
NS
NS
LP Velocity Fano Factor
Session Number
E
100s
20s
8s
4s
2s
1s
1s
x31
T6
T8
T12
T18
P22
MT
CRF5
CRF15
CRF30
CRF30
FR4
FR4
use of single correct forelimb
T6 0/16
T8 2/16
T12 11/16
T18 16/16
P22 16/16
F
% use of single correct forelimb
T6
T8
T12
T18
P22

Figure S1. A task for assessment of single individual forelimb movements, Related to Figure 1. A) Number of lever presses/minute. **B)** Number of performed sequences/minute. **C)** Fano Factor of the number of presses/sequence **D)** Fano Factor of the average velocity of lever press. Details on Table S1. **E)** Frames corresponding to the moment of lever press were identified for 5 specific session of the training schedule (Training days – T6, T8, T12 and T18 and Performance session – P22). Sixteen (16) randomly selected frames corresponding to 16 lever presses (or all, if less) were subsampled and visually inspected to assess the use of the correct forelimb. Data from one example animal is provided. **F)** Data from the 8 mice is summarized. In the last sessions near all presses (97.66% ± 2.34) were performed by the correct forelimb.

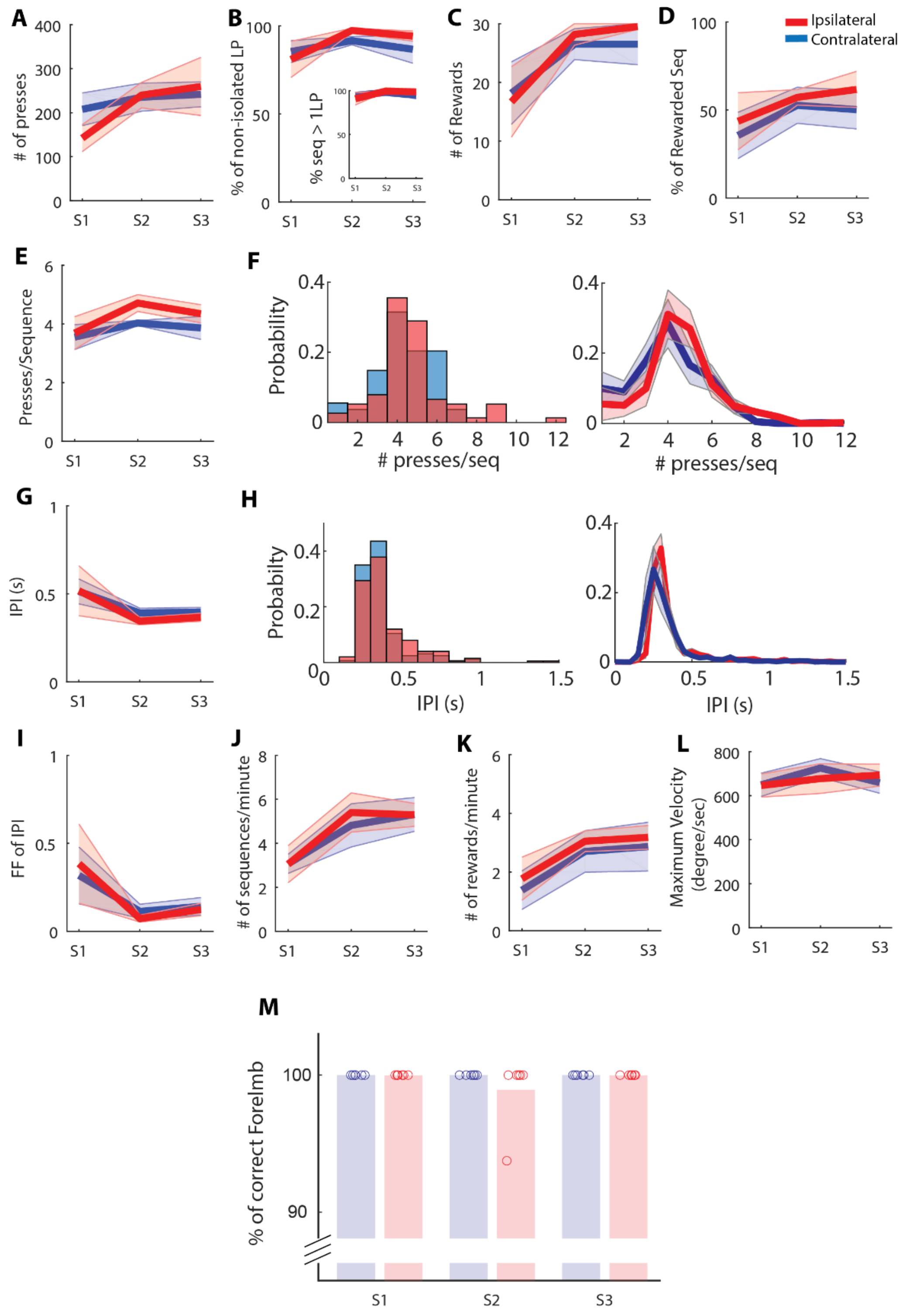

Figure S2. Mice perform the task using either the ipsi and contralateral forelimbs, Related to Figures 2, 3 and 4. Behavioral results from the 6 mice used in the analysis described in Figures

2-4. Data from sessions included in analysis in Figure 2 and Figure 3. Statistical details on Table S1. **A)** Total number of presses/session, **B)** % of sequences composed by more than one lever press - Inset % non isolated lever presses **C)** Number of Presses/Sequence. **D)** Histogram of the distribution of number of presses/sequence for ipsi and contralateral movements for one example animal (left) and for all animals across one day (right). **E)** Number of Rewards/Session **F)** % of rewarded Sequences. **G)** InterPress Intervals. **H)** Histogram of the distribution of IPIs for ipsi and contralateral movements for one example animal (left) and for all animals across one day (right) **I)** Fano Factor of InterPress Intervals **J)** Number of sequences performed/minute **K)** Number of rewards obtained/minute. **L)** Maximum velocity per press. **M)** % of correct forelimb use.

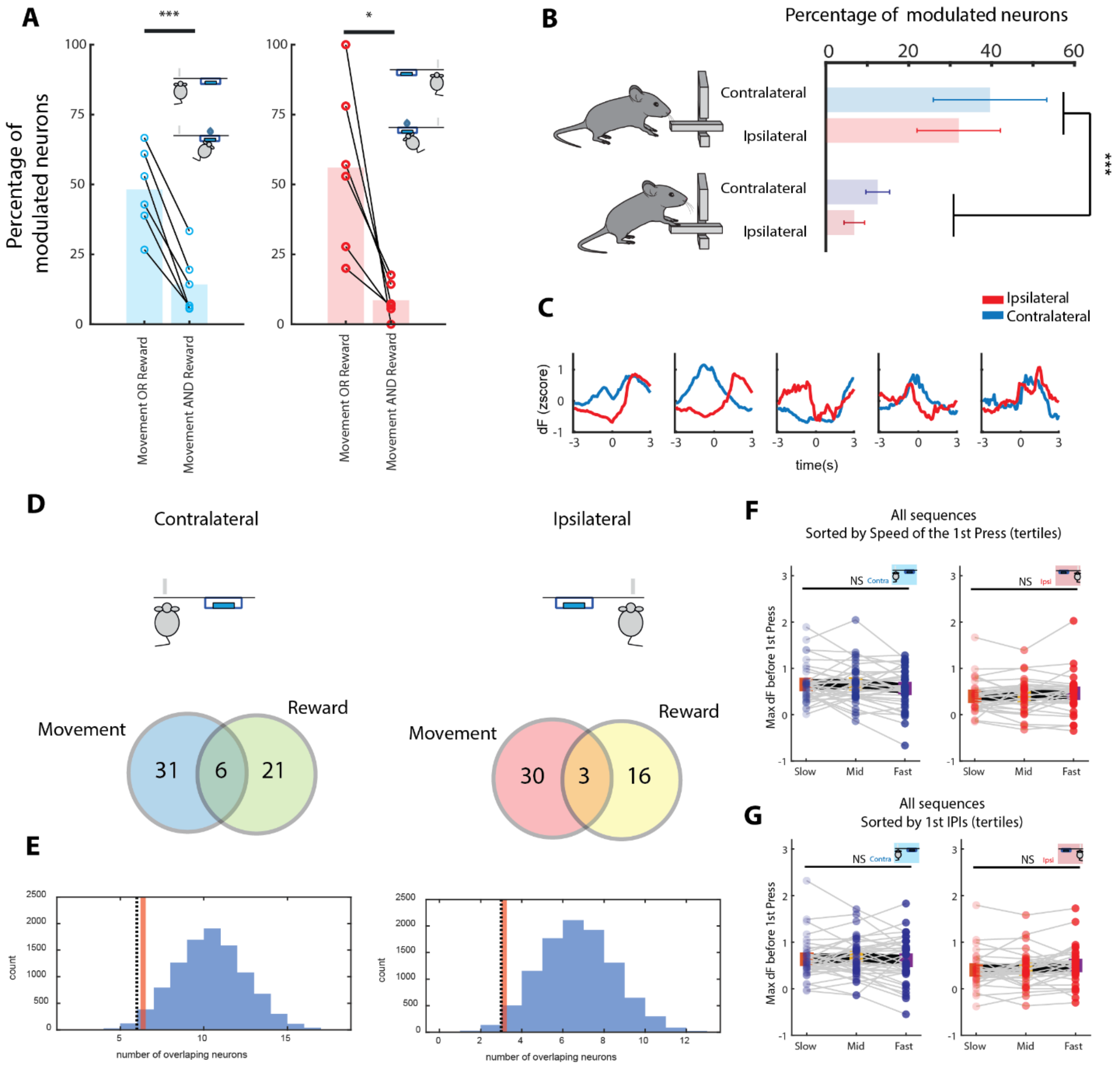

Figure S3. Modulation of activity of DANs by different events and considering different behavioral dimensions, Related to Figures 2 and 3. A) SNc DANs are modulated by one event. Neurons are more commonly modulated by only one event (movement or reward) than the two events (Left: 48,17 ± 6.07 vs. 14.21 ± 4.45, t=8.743, df=5, p<0.001; Right: 55.99 ± 12,28 vs 8.58 ± 2.60, t=3.576, df=5, p=0.0159) **B)** Activity before movement onset is more common that during execution. Number of neurons whose modulation started before movement sequence initiation (compare with Figure 2) and during sequence execution. Two-way repeated-measures ANOVA; main effect before/after F(1,5)=17.33, p=0.0088; main effect side F(1,5)=0.3986, p=0.5555; interaction effect F(1,5)=0.008, p=0.9336 **C)** Example of matched ROIs aligned to first lever press when action is performed by contralateral and ipsilateral forelimb. **D)** Overlap between reward-modulated neurons and movement initiation neurons is minimal and lower than expected by random allocation. **E)** Monte Carlo simulations (10,000 samples) were used to generate a distribution of the number of overlapping neurons for first press and reward, assuming random

assignment. Red line denote the lower margin of the one-sided 95% confidence interval of this simulation (upper limit is $+\infty$). Dashed line represents the number of overlapping neurons found in our experiments. **F)** Activity of movement modulated neurons, sorted by tertiles of the speed of the 1st press in the sequence for either ipsi ($F(2,72)= 1.557$, $p=0.2178$) or contralateral ($F(2,64)=1.127$, $p=0.335$) conditions. **G)** Activity of movement modulated neurons, sorted by tertiles of the duration of the 1st Interpress Interval for either ipsi ($F(2, 72)=0.3528$, $p=0.7039$) and contralateral ($F(2,64)=1.941$, $p=0.1519$) conditions.

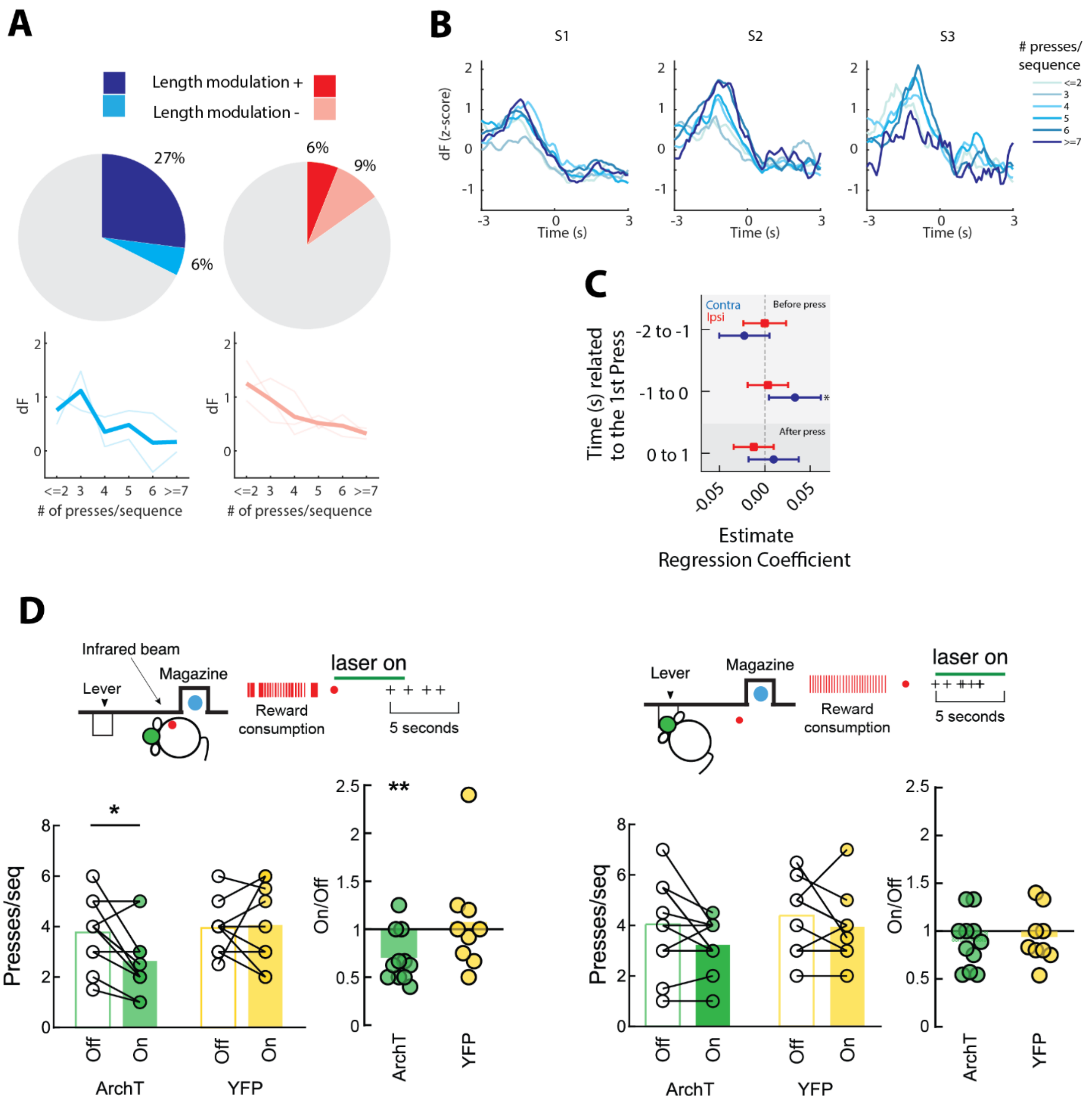

Figure S4. Transient SNc activity before the first lever press encodes the length of contralateral movement sequences, Related to Figures 3 and 5. A) Number of negatively modulated neurons in the ipsi and contralateral conditions. **B)** Activity of one example neuron (the one presented in Figure 3B) in Session 1, 2 and 3 sorted by number of presses/sequence in the contralateral sequence performance. **C)** A set of linear regression models were built to predict the number of presses based on trial-by-trial neural activity (adjusted for the neuron and mouse identity). When activity is considered in a trial-by-trial basis, only activity 1 second before first press of the contralateral sequences is related to the sequence length (R^2=0.158). **D)** Optogenetic inhibition of SNc DA neurons before movement initiation leads to a reduction in the length of movement sequences in a FR8 task. Left – Inhibition before sequence initiation leads to a

significant reduction in the number of presses in sequence (planned comparison ON/OFF ArchT: $t(10)=3.023$, $p=0.0128$; YFP: $t(8)=0.2088$, $p=0.8398$). When light-ON results were normalized to light-OFF results, there was a trend to a difference between groups: $U=24$, $p=0.0502$, with a significant difference from 1 (no change) in the ArchT group: $t(10)=3.707$, $p=0.004$, but not in the YFP group ($t(8)=0.4123$, $p=0.6909$). If inhibition was started after first press, no effect was seen neither in ArchT mice ($t(10)=2.043$, $p=0.0683$) or YFP mice ($t(8)=0.9363$, $p=3765$). When the results were normalized, there was no difference between groups ($U=46.50$, $p=0.8371$) and neither groups was significantly different from 1 (ArchT: $t(10)=1.300$, $p=0.2229$, YFP: $t(8)=0.6517$, $p=0.5329$)

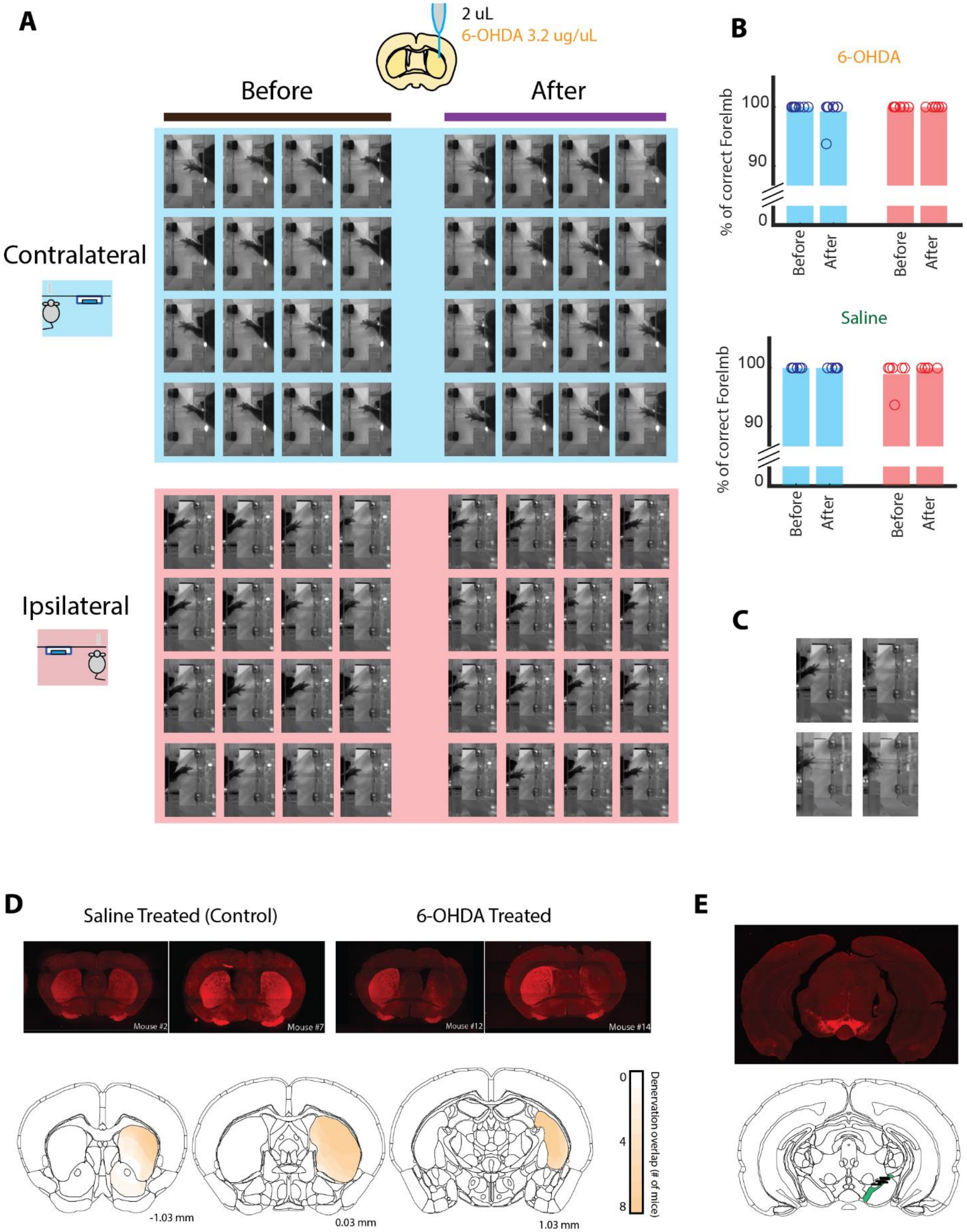

Figure S5. Mice solve the task using the forelimb contralateral to 6-OHDA lesion, **Related to Figure 5. A)** Still images from randomly selected lever presses of an example 6-OHDA treated mouse. Sixteen (16) randomly selected lever lever presses are used as an example in the 4

conditions (ipsi/contralateral forelimb and before/after 6-OHDA lesion). The mouse used the experiment-intended forelimb in all situations. **B)** For each condition and each animal 16 stills from randomly selected lever presses were visually inspected to assess the use of the correct. Two mice performed 1/16 (6.25%) of the inspected lever presses/condition with the incorrect forelimb (one 6-OHDA treated mouse in the contralateral forelimb after lesion and one saline treated mouse in the ipsilateral forelimb before). Percent of usage of the correct forelimb did not significantly changed after 6-OHDA lesion (Contra: 100% ± 0% to 99.21% ± 0.78; Ipsi: 100% ± 0 to 100% ± 0) or saline injection (Contra: 100% ± 0% to 100% ± 0; Ipsi: 98.96% ± 0.90 to 100% ± 0). **C)** The two situations with incorrect forelimb used are represented on the right images. An example of the same mouse using the correct forelimb is provided for comparison (left). **D)** Top Left: Tyrosine Hydroxylase staining of 2 example mice treated with 2 uL of intrastriatal saline (control group of Figure 5). Top Right: Tyrosine Hydroxylase staining of 2 example mice treated with 2 uL (3.2 ug/uL) of intrastriatal 6-OHDA (lesioned group in Figure 5). Bottom: Schematics representing overlap of 6-OHDA lesion across the 8 mice. Images were rotated as necessary to display the overlap of lesions in the same hemisphere (part of the animals were treated in the left striatum and the other part in the right). **E)** Tyrosine Hydroxylase staining of one of the mice with lens implant (left). Location of implanted lenses in 5 mice. Bars denote approximate position of lens tip. Lens trajectory was not possible to properly assess in one mouse due to severe tissue damage during perfusion.

**Fig**	**Sample size (n)**	**Statistical tests**	**Value**
1B	Wild-type (n=8)	One-way repeated measures ANOVA	Learning: **** F (18, 126) = 4,536 p<0.0001
			Performance: F (30, 210) = 1,174 p=0.2544
1C	Wild-type (n=8)	Mixed Effects	Learning: **** F (18, 109) = 7,526, p<0.0001<br>Learning Inset: **** F (18, 109) = 8.856, p<0.0001
			Performance: F (30, 200) = 1,283, p=0.1605<br>Performance Inset: F (30, 200) = 1,359, p=0.1121
1D	Wild-type (n=8)	Mixed Effect	L: F (18, 109) = 0.7912, p=0.7065
			P: * F (30, 200) = 1.642, p=0.0247
1E	Wild-type (n=8)	Mixed Effect	L: **** F (18, 109) = 10.52 p<0.0001
			P: F (30, 200) = 1,191, p=0.2383
		One sample t-test (vs. 4)	t7=0.5169, p=0.6212
1I	Wild-type (n=8)	Mixed Effect	L: ** F (18, 108) = 2,134, p=0.0089
			P: F (30,207) = 1.015, p=0.4507
		One sample t-test (vs. 1/3)	t7=0.3619, p=0.7281
1J	Wild-type (n=8)	Mixed Effect	L: F (18, 108) = 0.8126, p=0.6819
			P: * F (30, 200) = 1.736, p=0.0142
1K	Wild-type (n=8)	Mixed Effect	L: **** F (18, 109) = 3,250. p<0.0001
			P: F (30, 200) = 1.309, p=0.1423
2J	% of Positively modulated neurons (n=6)	Paired t-test	t=0.357, df=5, p=0.7356
2K	Positively modulated neurons:<br>Contra: n=37<br>Ipsi: n=33	Unpaired t-test	t=2.014, df=68, *p=0.0480
3A	Positively modulated neurons. Contra: n=37: Ipsi: n=33	Mixed effects analysis<br>Fixed effect<br>Test for Linear trend<br>Slope of linear fit	Contra:<br>F (5, 168) = 4.235, p=0.0012<br>**** F (1, 168) = 20.65, p<0.0001<br>Slope (95% CI): 0.0639 (0.0361 – 0.0916)<br>Ipsi:

			F (5, 151) = 1.470, p=0.2030<br>F (1, 151) = 0.4521 p=0.5023<br>Slope (95% CI): -0.0086 (-0.0347 – 0.0170)
3C	Length modulated neurons.	Fisher Exact test	* Contra: n=10/37: Ipsi: n=2/33; p=0.0266
3F	ROIs correlation n=114	One-way repeated measures ANOVA<br>Tukey Post-hoc test	F(1.353, 152.8) = 155.1 p<0.0001<br>*** Matched vs. Different: p<0.001<br>*** Matched vs. Shuffled: p<0.001
3G	Positively modulated neurons. Contra: n=37: Ipsi: n=33	Mixed effects analysis<br>Fixed effect<br>Test for Linear trend<br>Slope of linear fit	Contra:<br>F (5, 175) = 6.207, p<0.0001<br>**** F (1, 175) = 24.69, p<0.0001<br>Slope (95% CI): 0.0607 (0.0366 – 0.0848)<br>Ipsi:<br>F (5, 157) = 1.038, p=0.2913<br>F (1, 157) = 1.202 p=0.2470<br>Slope (95% CI): 0.0112 (-0.0099 – 0.0345)
3H	Length modulated neurons.	Fisher Exact test	* Contra: n=9/37: Ipsi: n=1/33; p=0.0151
4C	% of Positively modulated neurons (n=6)	Paired t-test	t=0.2668, df=5, p=0.8003
4F Left	% of Positively modulated neurons (n=6)	Paired t-test	t=0.1452. df=5, p=0.8902
4F Right	Positively modulated neurons<br>Contra: n=27<br>Ipsi: n=19	Unpaired t-test	t=0.974, df=44, p=0.3355
4G Left	% of Positively modulated neurons (n=6)	Paired t-test	t=0.1156, df=5, p=0.9125
4G Right	Positively modulated neurons<br>Contra: n=12<br>Ipsi: n=22	Unpaired t-test	t=2.723, df=32, p=0.0104
5C left	Presses/sequence (n=8)	Repeated measures two-way ANOVA	**** Time: F (1, 7) = 68.90 P<0.0001<br>Forelimb: F (1, 7) = 4.704 p=0.0667

			* Time x Forelimb: F (1, 7) = 11,11 p=0.0125<br>Sidak's multiple comparison test<br>*** Contralateral p=0.0005<br>Ipsilateral p=0.1380
5C right	Normalized presses/sequence (n=8)	Paired t-test	** t=3,759, df=7 p=0.0071
		One sample t-test	**** Contralateral: t=11.07, df=7 p<0.0001<br>Ipsilateral: t=2.281, df=7 p=0.0565
5D left	Presses/sequence (n=6)	Repeated measures two-way<br>ANOVA	* Time: F (1, 5) = 7.704 p=0.0391<br>Forelimb: F (1, 5) = 2.007 p=0.2157<br>Time x Forelimb: F (1, 5) = 0.01041 p=0.9227
5D right	Normalized presses/sequence (n=6)	Paired t-test	t=0.4441, df=5, p=0.6755
5E left	% of Long sequences (n=8)	Repeated measures two-way ANOVA	*** Time: F (1, 7) = 30.12 p=0.0009<br>Forelimb: F (1, 7) = 2.087 p=0.1918<br>*** Time x Forelimb: F (1, 7) = 32.45 p=0.0007<br>Sidak's multiple comparison test<br>*** Contralateral p=0.0003<br>Ipsilateral p=0.9725
5E right	Change in long sequences (n=8)	Paired t-test	** t=4,126, df=7 p=0.0044
		One sample t-test	**** Contralateral: t=15,46, df=7 p<0.0001<br>Ipsilateral: t=0.2100. df=7 p=0.8397
5F left	% of Long sequences (n=6)	Repeated measures two-way ANOVA	Time: F (1, 5) = 4.911 p=0.0775<br>Forelimb: F (1, 5) = 0.8281 p=0.4046<br>Time x Forelimb: F (1, 5) = 0.003268 p=0.9566
5F right	Change in long sequences (n=6)	Paired t-test	t=0.5099, df=5 p=0.6319
S1A	Wild-type (n=8)	Repeated Measures ANOVA	**** L: F (18, 126) = 4.254, p<0.0001<br>**P: F (30, 210) = 2.088, p=0.0.0025
S1B	Wild-type (n=8)	Repeated Measures ANOVA	** L: F (18, 126) = 2.569, p=0.0012<br>** P: F (30, 210) = 2.006 p=0.0025
S1C	Wild-type (n=8)	Mixed Effect	* L: F (18, 109) = 1.928, p=0.0205<br>P: F (30, 200) = 1.317, p=0.1371
S1D	Wild-type (n=8)	Mixed Effect	L: F (18, 109) = 1.692, p=0.0513<br>P: F (30, 200) = 1.082, p=0.3610
S2A	Animal (n=6)	Repeated measures two-	Session: F(2,10) = 1.330, p=0.3075

		way ANOVA	Side: F(1,5)=0.2092, p=0.6666<br>Session x Side: F(2,10)=1.290, p=0.3175
S2B	Animal (n=6)	Repeated measures two-way ANOVA	Session: F(2,10) = 1.032, p=0.3914<br>Side: F(1,5)=0.03333, p=0.8623<br>Session x Side: F(2,10)=1.094, p=0.3719
S2B (inset)	Animal (n=6)	Repeated measures two-way ANOVA	Session: F(2,10) = 1.219, p=0.3358<br>Side: F(1,5)=0.3852, p=0.5620<br>Session x Side: F(2,10)=0.8604, p=0.4521
S2C	Animal (n=6)	Repeated measures two-way ANOVA	Session: F(2,10) = 6.091, p=0.0186<br>Side: F(1,5)=0.05358, p=0.8261<br>Session x Side: F(2,10)=0.2880, p=0.7558
S2D	Animal (n=6)	Repeated measures two-way ANOVA	Session: F(2,10) = 2.179, p=0.1640<br>Side: F(1,5)=0.3660, p=0.5716<br>Session x Side: F(2,10)=0.07044, p=0.9324
S2E	Animal (n=6)	Repeated measures two-way ANOVA	Session: F(2,10) = 1.985, p=0.1880<br>Side: F(1,5)=2.919, p=0.1483<br>Session x Side: F(2,10)=0.4165, p=0.6703
S2G	Animal (n=6)	Repeated measures two-way ANOVA	Session: F(2,10) = 2.009, p=0.1847<br>Side: F(1,5)=0.3257, p=0.5929<br>Session x Side: F(2,10)=0.4805, p=0.6320
S2I	Animal (n=6)	Repeated measures two-way ANOVA	Session: F(2,10) = 1.513, p=0.2667<br>Side: F(1,5)=0.004525, p=0.9490<br>Session x Side: F(2,10)=0.9085, p=0.4340
S2J	Animal (n=6)	Repeated measures two-way ANOVA	Session: F(2,10) = 3.992, p=0.0532<br>Side: F(1,5)=0.08217, p=0.7859<br>Session x Side: F(2,10)=0.1292, p=0.8803
S2K	Animal (n=6)	Repeated measures two-way ANOVA	Session: F(2,10) = 3.912, p=0.0556<br>Side: F(1,5)=0.2349, p=0.6484<br>Session x Side: F(2,10)=0.001934, p=0.9981
S2L	Animal (n=6)	Repeated measures two-way ANOVA	Session: F(2,10) = 4.075, p=0.0508<br>Side: F(1,5)=0.03228, p=0.8645<br>Session x Side: F(2,10)=0.6672, p=0.5346

Table S1. Detailed statistical analysis, Related to Figures 1, 2, 3, 4 and 5, Figures S1 and S2.
